# Bioactive-Rich *Piper sarmentosum* Aqueous Extract Mitigates Osteoarthritic Pathology by Enhancing Anabolic Activity and Attenuating NO-Driven Catabolism in Human Chondrocytes

**DOI:** 10.3390/biomedicines14010128

**Published:** 2026-01-08

**Authors:** Yi Ting Lee, Mohd Heikal Mohd Yunus, Rizal Abdul Rani, Chiew Yong Ng, Muhammad Dain Yazid, Azizah Ugusman, Jia Xian Law

**Affiliations:** 1 Department of Physiology, Faculty of Medicine, Universiti Kebangsaan Malaysia, Cheras, Kuala Lumpur 56000, Malaysia; 2 Department of Orthopaedics and Traumatology, Faculty of Medicine, Hospital Canselor Tuanku Muhriz, Universiti Kebangsaan Malaysia, Cheras, Kuala Lumpur 56000, Malaysia; 3 Department of Tissue Engineering & Regenerative Medicine, Faculty of Medicine, Universiti Kebangsaan Malaysia, Cheras, Kuala Lumpur 56000, Malaysia

**Keywords:** *Piper sarmentosum*, osteoarthritis, chondrocytes, chondroprotective, anti-inflammatory, antioxidant, nitric oxide signaling

## Abstract

**Background:** Osteoarthritis (OA) is a prevalent degenerative joint disease often causing functional disability. Current therapies provide only temporary relief and can cause adverse effects that frequently result in pain and disability. Current pharmacological options offer only temporary symptom relief and may cause adverse effects. *Piper sarmentosum* (PS), a plant traditionally used for its medicinal properties, has demonstrated antioxidant and anti-inflammatory activities that may counteract OA-related degeneration. This study provides preliminary insight into the therapeutic potential of PS aqueous extract in human OA chondrocytes. **Methods:** Compounds in the PS aqueous extract were profiled using liquid chromatography–tandem mass spectrometry (LC-MS/MS). Primary human OA chondrocytes (HOCs) were treated with 0.5, 2, and 4 µg/mL of PS aqueous extract for 72 h. Key OA-related parameters were assessed, including anabolic markers (sulfated glycosaminoglycan (sGAG), collagen type II (COL II), aggrecan core protein (ACP), SRY-box transcription factor 9 (SOX9)), catabolic markers (matrix metalloproteinase (MMP) 1, MMP13, cyclooxygenase 2 (COX2)), oxidative stress (nitric oxide (NO) production, inducible NO synthase (iNOS) expression), and inflammatory responses (interleukin (IL) 6). Gene expression was quantified using qPCR, and protein levels were evaluated using the colorimetric method, immunocytochemistry, and Western blot. **Results:** A total of 101 compounds were identified in the extract, including vitexin, pterostilbene, and glutathione—bioactives known for antioxidant, anti-inflammatory, and chondroprotective functions. PS-treated chondrocytes maintain healthy polygonal morphology. PS aqueous extract significantly enhanced anabolic gene expression (COL2A1, ACP, SOX9) and sGAG production, while concurrently suppressing COX2 expression and NO synthesis. Additionally, PS aqueous extract reduced COX2 and iNOS protein levels, indicating inhibition of the NO signaling pathway. Catabolic activity was attenuated, and inflammatory responses were partially reduced. **Conclusions:** PS aqueous extract exhibits promising chondroprotective, antioxidant, and anti-inflammatory effects in human OA chondrocytes, largely through the suppression of NO-mediated catabolic signaling. The presence of multiple bioactive compounds supports its mechanistic potential. These findings highlight PS aqueous extract as a potential therapeutic candidate for OA management. Further ex vivo and in vivo studies are warranted to validate its efficacy and clarify its mechanism in joint-tissue environments.

## 1. Background

Osteoarthritis (OA) is a chronic medical condition caused by abnormalities in the cartilage and subchondral bone of joints, leading to disability [1,2]). According to the World Health Organization (WHO), OA affected 528 million people globally in the year 2019 [3] From 1990 to 2019, the number of OA cases increased worldwide by 113.25%, from 247.51 million to 527.81 million cases [4] OA often progresses slowly and persistently over a prolonged period [5]. It usually affects the joints and adjacent tissues necessary for movement, such as the articular cartilage, ligament, meniscus, tendons, synovium, and bones [6]. Although OA can affect any joint in the body, it is more frequently observed in large weight-bearing joints, including the knees and hips [7].

The progression of OA is intricate and involves the interplay of various factors, including alterations in mechanical loading, injury, genetic predispositions, and shifts in the articular chondrocyte gene expression patterns [8,9]. Alterations in structural and metabolic processes in articular cartilage are believed to be pivotal in the initiation and development of OA [10], particularly when the chondrocytes lose their ability to achieve a balance between the degradation and synthesis of the extracellular matrix (ECM) [11]. This is because articular cartilage consists of only a single cell type, chondrocytes, and it is aneural, avascular, and alymphatic. Hence, any alteration in this composition will affect the homeostasis of cartilage [12].

The ECM of healthy articular cartilage is mainly composed of collagen type II (COL II), which provides tensile strength, and aggrecan, which contributes to stiffness and compression [13]. The progressive loss of articular cartilage and matrix degradation caused by the proteolysis of collagen and proteoglycans are the hallmarks of OA [10]. As a result, fibroblastic markers such as collagen type I (COL I) and X (COL X) are expressed more frequently due to chondrocytes’ dedifferentiation, and the expression of chondrocyte markers such as aggrecan, COL II, and SRY-box transcription factor 9 (SOX9) decreases [14]. Moreover, pro-inflammatory cytokines such as interleukin (IL)-1 and IL-6 are upregulated as OA progresses. The secretion of IL-1 triggers the release of other inflammatory cytokines, including tumor necrosis factor (TNF)-α, IL-6, and IL-8, as well as various matrix metalloproteinases [15,16]. Moreover, IL-1 also stimulates the production of prostaglandin (PG) E2, resulting in the formation of osteophytes and nitric oxide (NO), which can lead to oxidative damage and cell death [17,18]. Together, these cytokines inhibit the synthesis of ECM and cause the destruction of cartilage in joints [10].

Currently, there are various treatments available for treating OA. However, there is no proven treatment to halt or reverse OA progression. The main objectives of currently available OA management are to improve overall quality of life, minimize disability, and relieve pain [19]. These include nonpharmacological management methods such as weight management, self-management, exercise and strength training, pharmacological management using nonsteroidal anti-inflammatory drugs (NSAIDs), vicosupplementation, arthroscopic surgery, and total knee replacement [19,20,21,22]. The use of NSAIDs offers substantial benefits in OA treatment. Nonetheless, the long-term use of these drugs is associated with negative effects on the kidneys, cardiovascular system, and gastrointestinal system [23,24]. Symptomatic end-stage OA is normally treated with total knee replacement. Total knee replacement is a successful intervention, but the functional outcomes may be less than ideal, and the longevity of prosthetic devices is limited [8]. Therefore, the development of disease-modifying OA drugs (DMOADs) that can stop or reverse the progression of OA with minimal detrimental effects is crucial.

Given the adverse outcomes associated with OA medications, researchers are further exploring the natural substances that exhibit chondroprotective effects. Numerous studies have determined the effects of natural compounds on OA and have proven that natural compounds such as resveratrol, berberine, and wogonin demonstrate chondroprotective and osteoprotective effects. These compounds serve as anti-inflammatory and antioxidant agents, help regulate chondrocyte catabolism, inhibit osteoclast differentiation, and promote ECM synthesis [5,25]. Moreover, the minimal or nonexistent side effects of natural compounds render them safer options for the treatment of OA [26].

In this study, *Piper sarmentosum* (PS) aqueous extract was studied to elucidate its effect on human OA articular cartilage. It is an herbaceous plant from the family Piperaceae that is commonly found in Southeast Asian countries and the Southeast coastal regions of China. Traditionally, it has been used for treating fever, toothache, indigestion, abdominal distension, anorexia, skin diseases, and rheumatic bone pain [27]. According to recent research, PS aqueous extract has several protective effects, such as antihypertensive, anti-inflammatory, antibacterial, anti-neoplastic, antioxidant, anti-allergic, anti-atherosclerotic, antidiabetic, and antidepressant effects [28,29,30,31,32,33,34,35,36]. The potential anti-inflammatory and antioxidant effects of PS might be due to high concentrations of amide alkaloids and flavonoids such as sarmentine, sarmentosine, and naringenin [37,38,39]. The study performed by Phong et al. [40] showed that the amide alkaloids that consist of *Piper* species can inhibit NO production. In comparison, flavone shows antioxidant properties and is known to reduce inflammation by inhibiting inflammatory mediators such as PGE2 and NO [41]. Despite these features, no study has been conducted to determine the potential of PS for treating OA. This inspired us to explore the potential of PS in the treatment of OA. Therefore, the aim of the present study was to investigate the effect of PS aqueous extract on the anabolic and catabolic activity of human OA chondrocytes in vitro, which may provide useful information for the management of OA.

## 2. Methods

### 2.1. Preparation of PS Aqueous Extract

The PS aqueous extract powder was procured from HERBagus Sdn. Bhd., Penang, Malaysia, with a single batch used throughout the study. To prepare the PS extract, the aerial parts of the plants were mixed with pure water and subjected to extraction at 100 °C for 3 h. Following extraction, the mixture was filtered, and the resulting filtrate was concentrated at 60 °C for 3 h. Finally, the concentrated extract was dried using a spray dryer to produce the PS extract powder. The extract was stored in a −20 °C freezer until further use.

### 2.2. Liquid Chromatography-Tandem Mass Spectrometry (LC-MS/MS)

Sample separation was performed using a Thermo Scientific C18 column (AcclaimTM Polar Advantage II, 3 × 150 mm, 3 µm particle size) (Thermo Fisher Scientific, Waltham, MA, USA) on Bruker Elute Plus UHPLC (Bruker Corperation, Bremen, Germany), Pump HPG 1300. Gradient elution was performed at a flow rate of 0.4 mL/min and 40 °C column temperature using H_2_O + 0.1% Formic Acid (A) and 100% Acetonitrile (ACN) (B) with a 22 min total run time. The injection volume of the sample was 5 µL. The gradient started at 5% B (0–3 min); 80% B (3–10 min); 80% B (10–15 min), and 5% B (15–22 min). After that, mass spectrometry was carried out using a TimsTOF Pro 2 Bruker Daltonics (Bruker Daltonics Inc., Billerica, MA, USA) equipped with Electrospray Ionization (ESI) in the positive mode. The accurate mass data of the molecular ions, provided by the time-of-flight (TOF) analyzer, were processed by Compass Data Analysis software version 6.1 (Bruker Daltonik, Bremen, Germany. Detected compounds were ranked based on their signal-to-noise (S/N) ratios as an estimate of detection quality and relative abundance. The top 20 compounds with the highest S/N values were selected for further discussion.

### 2.3. Human OA Articular Chondrocyte Isolation and Culture

Prior ethical approval was obtained from the Research Ethics Committee, Universiti Kebangsaan Malaysia (UKM) (UKM PPI/111/8/JEP-2022-136). Human OA articular cartilage samples were obtained from consented patients aged 55–70 years who were undergoing total knee arthroplasty (TKR) at the Hospital Canselor Tuanku Muhriz (HCTM), Kuala Lumpur, Malaysia. Each patient was identified with knee OA, with lesions categorized as grade 4 in accordance with the International Cartilage Repair Society (ICRS) scoring. All the samples were processed within 24 h of the surgery by removing the articular cartilage from the distal femur’s medial and lateral condyles. The specimens were washed and minced into small fragments. The samples were then digested with 0.6% collagenase type II (Worthington Biomedical, Lakewood, CO, USA) in an S150 orbital incubator (Stuart Scientific, Stone, UK). The cells were then cultured in 6-well plates (NEST, Wuxi, China) with Nutrient mixture F-12 and Dulbecco’s Modified Eagle Medium (FD; Gibco, Thermo Fisher Scientific, Waltham, MA, USA) complete (FDC) consisting of FD, 10% fetal bovine serum (FBS; Capricorn Scientific, Ebsdorfergrund, Germany), and 1% antibiotic-antimycotic (AA; Capricorn Scientific, Ebsdorfergrund, Germany). Once the cells were attached to the plate and spread, the culture medium was changed to chondrocyte growth medium (PromoCell, Heidelberg, Germany). After reaching 80% confluency, the chondrocytes were trypsinized and subcultured in new 6-well plates. Once the cells reached 80% confluence, the chondrocytes were treated with different concentrations of PS aqueous extract in FD basal medium.

#### 2.3.1. 3-(4,5-Dimethylthiazol-2-yl)-2,5-Diphenyltetrazolium Bromide (MTT) Assay

In general, chondrocytes were cultured at a density of 25,000 cells/cm^2^ in 96-well plates and allowed to attach. After 24 h, treatment media containing 0.5–16 µg/mL PS aqueous extract were added. To assess the cell viability, an MTT assay was conducted on days 1, 3, and 5 post-treatment. The treatment media was removed, and the plates were rinsed with Dulbecco’s Phosphate-Buffered Saline (DPBS). Then, FD basal medium supplemented with 5 mg/mL MTT solution was added to each well and incubated at 37 °C for 4 h. Following incubation, the solution was removed, and the formazan crystals were solubilized with dimethyl sulfoxide (DMSO; Sigma-Aldrich, St. Louis, MO, USA). The absorbance was read using a spectrophotometer (BioTek, PowerWave XS, Highland Park, IL, USA) at 570 nm.

#### 2.3.2. Quantitative Real-Time Polymerase Chain Reaction (qPCR)

Total ribonucleic acid (RNA) was collected for the measurement of gene expression between different groups using a RNeasy^®^ Mini Kit (Qiagen Inc., Germantown, PA, USA) according to the manufacturer’s instructions. Subsequently, a QuantiTect^®^ Reverse Transcription Kit (Qiagen Inc., Germantown, PA, USA) was used to produce complementary deoxyribonucleic acid (cDNA) from the total RNA through a reverse transcription process. Then, the gene expression of catabolic and anabolic markers of OA chondrocytes, including matrix metalloproteinase (MMP) 1, MMP13, IL-6, COX2, iNOS, SOX9, ACP, COL I, and COL II, was determined using QuantiNova^TM^ SYBR^®^ Green PCR Kit (Qiagen Inc., Germantown, PA, USA). Both the forward and reverse primers were synthesized using Primer3 software based on the sequences published in GenBank (Table 1). The housekeeping gene used was the human glyceraldehyde-3-phosphate dehydrogenase (GAPDH). The reaction mixture was prepared according to the manufacturer’s instructions. The PCRs were run for 40 cycles in a CFX96 Real-Time PCR Detection System (Bio-Rad Laboratories, Hercules, CA, USA) as follows: initial heat activation for 2 min at 95 °C, denaturation step for 5 s at 95 °C, and combined annealing/extension for 10 s at 60 °C. The 2(−∆∆Ct) method was employed to analyze the relative messenger RNA (mRNA) expression, with normalization to the GAPDH gene.

#### 2.3.3. Nitric Oxide (NO) Assay

Total Nitric Oxide and Nitrate/Nitrite Assay kit (Parameter, R&D Systems Inc., Minneapolis, MN, USA) was used to determine the total NO level in the samples based on the measurement of the stable metabolites of NO, which are nitrite (NO_2_^−^) and nitrate (NO_3_^−^). The cell culture supernatant was filtered using a 10,000 molecular weight cut-off (MWCO) filter (Amicon Ultra-4 Centrifugal Filters; Ultracel^®^, Merck Milipore Ltd., Carrigtwohill, Ireland), aliquoted, and stored at −20 °C to avoid repeated freeze–thaw cycles. The level of NO in the supernatant was determined by converting nitrate to nitrite using the enzyme nitrate reductase, followed by the production of chromophoric azo derivatives through the Griess reaction. The optical density was measured using a spectrophotometer (BioTek, PowerWave XS, Highland Park, IL, USA) at 540 nm with wavelength correction at 690 nm. A standard curve was plotted to determine the total nitrite and nitrate concentrations in the samples.

#### 2.3.4. Sulfated Glycosaminoglycan (sGAG) Assay

A Blyscan^TM^ Sulfated Glycosaminoglycan Assay Kit (Biocolor Ltd., Northern Ireland, UK) was used to measure the total sGAG content in the samples. In this study, both cell culture medium and cellular sGAG were collected to determine the total sGAG in a sample. The cellular sGAG was collected using papain extraction reagent, which was prepared according to the manufacturer’s protocol. This assay is based on a quantitative dye-binding method involving the use of 1, 9-dimethylmethylene blue, which binds specifically to the sGAG chains of the sulfated polysaccharide component of proteoglycans. After the formation of the sGAG-dye complex, the tubes were centrifuged to remove the unbound dye and dissociated with dissociation reagent and centrifuged again. Finally, 200 µL of each sample was transferred to a 96-well plate, and the absorbance was measured at 656 nm using a spectrophotometer. The sGAG concentration of each sample was obtained by plotting the standard curve.

#### 2.3.5. Immunocytochemistry (ICC)

After treatment, the cells were washed with DPBS, and then 4% paraformaldehyde (PFA; Sigma-Aldrich, USA) was used to fix the cells. Then, permeabilization was performed using a 0.5% Triton X-100 solution (Sigma-Aldrich, USA). The blocking process was performed with 10% normal goat serum (Agilent Technologies, Gaithersburg, MD, USA). After blocking, the cells were incubated with mouse monoclonal anti-β-actin antibody (AB8226, Abcam, Cambridge, MA, USA), rabbit monoclonal recombinant anti-iNOS antibody (AB178945; Abcam, Cambridge, MA, USA), rabbit monoclonal recombinant anti-COX2 antibody (AB179800, Abcam, Cambridge, MA, USA) and rabbit polyclonal anti-phospho-iNOS (phospho-Tyr151) antibody (Signalway Antibody LLC, College Park, MD, USA) at a dilution factor of 1:500 at 4 °C overnight. Then, the cells were incubated with goat anti-mouse IgG, Alexa Fluor^®^ 488 (Abcam, Waltham, MA, USA), and goat anti-rabbit IgG Alexa Fluor^®^ 594 (Abcam, Waltham, MA, USA) at a dilution factor of 1:1000. Next, the cells were rinsed and incubated with 1 μM 4′,6-diamidino-2-phenylindole (DAPI; Sigma-Aldrich, Burlington, VT, USA). Lastly, the cells were washed, and the stained cells were immersed in DPBS. Fluorescence images were obtained via Nikon AX Confocal Laser Microscope (Nikon, Tokyo, Japan) at a magnification of 20×.

#### 2.3.6. Cell Lysate Preparation

The cells were trypsinized and centrifuged. The resulting pellet was then lysed using radioimmunoprecipitation assay (RIPA) buffer (Thermo Fisher Scientific, Waltham, MA, USA) supplemented with 1% of Halt^TM^ protease and phosphatase inhibitor cocktail (Thermo Fisher Scientific, Waltham, MA, USA) and incubated on ice for 15 min. Subsequently, the protein lysate was centrifuged, and the supernatant was collected for Western blot analysis.

#### 2.3.7. Sodium Dodecyl Sulfate-Polyacrylamide Gel Electrophoresis (SDS-PAGE) and Western Blotting

The protein sample for SDS-PAGE was prepared by mixing with 5× loading dye with SDS (Elabscience, Wuhan, China). Subsequently, the samples, along with PageRuler^TM^ Prestained Protein Ladder (Thermo Fisher Scientific, Waltham, MA, USA) or BLUeye Prestained Protein Ladder (Bio-Helix Co., Ltd., New Taipei City, Taiwan), were loaded onto an in-house prepared 8% SDS-PAGE gel. Protein separation was performed using a Mini Gel tank (Invitrogen, Carlsbad, CA, USA). After the electrophoresis process, the proteins were transferred to a 0.2 µm nitrocellulose membrane (Bio-Rad, Hercules, CA, USA) using a semi-dry transfer system (Trans-Blot^®^ SD Cell, Bio-Rad, Hercules, CA, USA). Following the transfer process, the blocking step was carried out using 5% skim milk (Sigma-Aldrich, Burlington, VT, USA) in Tris-buffered saline with 0.1% Tween 20 (TBS-T; Elabscience, Houston, TX, USA) or 3% bovine serum albumin (BSA; Elabscience, Houston, TX, USA). After that, the samples were incubated overnight at 4 °C with constant shaking in diluted primary antibody. The primary antibodies used were rabbit anti-iNOS antibody (1:1000) (AB178945, Abcam, Cambridge, MA, USA), rabbit anti-COX2 antibody (1:1000) (AB179800, Abcam, Cambridge, MA, USA), rabbit anti-phospho-iNOS (phospho-Tyr151) (1:1000) (Signalway Antibody LLC, USA) and mouse anti-β actin antibody (1:1000) (AB8226, Abcam, Cambridge, MA, USA). The next day, the samples were incubated with rabbit IgG horseradish peroxidase (HRP)-conjugated antibody (1:5000) (Abcam, Cambridge, MA, USA) or mouse IgG HRP-conjugated antibody (1:5000) (Cell Signaling Technology, Danvers, MA, USA). Lastly, the membrane was incubated with Pierce^TM^ enhanced chemiluminescence (ECL) Western Blotting Substrate (Thermo Fisher Scientific, Waltham, MA, USA) and visualized using Amersham Imager 600 (GE Healthcare Life Sciences, Chicago, IL, USA).

#### 2.3.8. Statistical Analysis

Each group consisted of three biological samples from different patients to ensure reproducibility. Data analysis was performed using GraphPad Prism version 9.4.1 (GraphPad Software Inc., San Diego, CA, USA). The data are presented as mean ± standard error of the mean (SEM). The statistical analysis between the control and treatment groups was conducted using one-way or two-way analysis of variance (ANOVA). A result with a *p*-value less than 0.05 was considered statistically significant. In this study, the ICC was employed as a qualitative assessment tool.

## 3. Results

### 3.1. Identification of Bioactive Compounds in PS Aqueous Extract by LC-MS/MS

A total of 101 compounds were detected in the extract. The top 20 compounds with the highest S/N ratios were selected (Table 2), as these represent the most reliably detected and likely abundant constituents. Among the 20 most abundant compounds identified, 15 were documented in the literature to exhibit anti-inflammatory, antioxidant, or chondroprotective properties, which are pertinent to OA management. Three compounds lacked available scientific evidence of bioactivity, and two were known for their fragrance or aromatic properties but had no known therapeutic roles. These findings imply that the bioactivity observed in the in vitro assays may be partially attributed to the presence of these bioactive phytochemicals. The complete list of compounds found has been included in the Supplementary Material File S1.

### 3.2. Chondrocyte Viability

**Figure 1:**
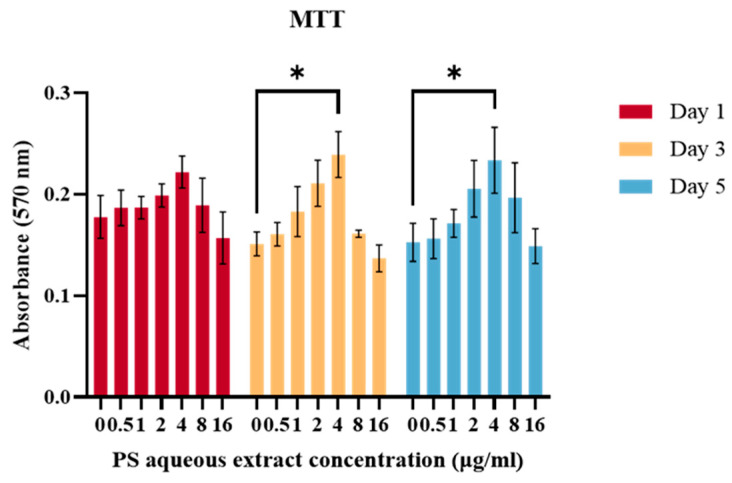
The viability of human OA chondrocytes treated with various PS aqueous extract concentrations. The values are expressed as the mean ± SEM, N = 3. * *p* < 0.05.

Figure 1 shows the viability of chondrocytes treated with different concentrations (0, 0.5, 1, 2, 4, 8, 16 µg/mL) of PS aqueous extract. There was no significant difference in the cell viability on days 3 and 5 when compared to that on day 1. This result suggested that the PS aqueous extract had no adverse effect on the viability of chondrocytes at these concentrations. Furthermore, the viability of the chondrocytes treated with 4 µg/mL was significantly greater (*p* < 0.05) than that of the untreated chondrocytes at days 3 and 5. Hence, for the subsequent experiments, 0.5, 2 and 4 µg/mL PS aqueous extract were used to treat the chondrocytes for a duration of three days in the subsequent experiments.

### 3.3. Chondrocytes Morphology

**Figure 2:**
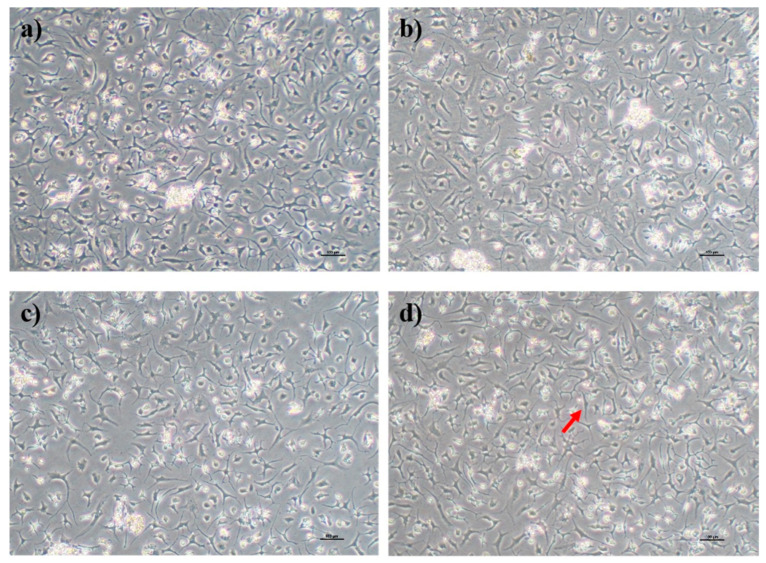
Morphology of human OA chondrocytes in monolayer culture. Human OA chondrocytes cultured in FD basal media (**a**), FD supplemented with 0.5 µg/mL PS aqueous extract (**b**), FD supplemented with 2 µg/mL PS aqueous extract (**c**), and FD supplemented with 4 µg/mL PS aqueous extract (**d**). The red arrow indicates that the OA chondrocytes had taken on a fibroblastic appearance. The cells were visualized under 40× magnification, and each scale bar represents 100 µm.

Human OA chondrocytes were cultured in FD media supplemented with or without PS aqueous extract for 3 days. A slight difference in morphology was observed as the concentration of the PS aqueous extract increased. The chondrocytes cultured in FD basal media and FD media supplemented with 0.5 and 2 µg/mL PS aqueous showed similar morphologies, with more polygonal-shaped cells and fewer dendrite-like features (Figure 2a–c). The chondrocytes cultured in media supplemented with 4 µg/mL PS aqueous extract demonstrated more dendrite-like features, a more fibroblastic morphology (red arrow in Figure 2), and loss of the polygonal shape of the chondrocytes (Figure 2d).

### 3.4. The Effects of PS Aqueous Extract on Anabolic Gene Expression

**Figure 3:**
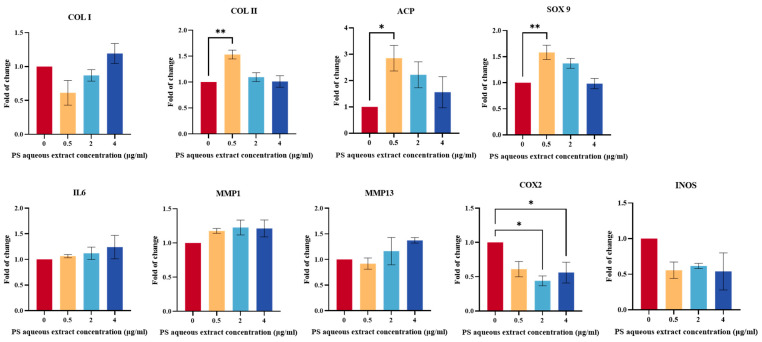
Anabolic and catabolic gene expression of human OA chondrocytes treated with different PS aqueous extract concentrations. The values are expressed as the mean ± SEM, N = 3. * *p* < 0.05, ** *p* < 0.01. Collagen type I, COL I; collagen type II, COL II; aggrecan core protein, ACP; SRY-box transcription factor 9, SOX9; Interleukin 6, IL6; matrix metalloproteinase, MMP; cyclooxygenase 2, COX2; inducible nitric oxide synthase, iNOS.

The gene expression of anabolic factors (COL I, COL II, ACP, and SOX9) is important for promoting ECM secretion and modulating tissue remodeling. As shown in Figure 3, there was no significant difference in the gene expression of COL I in human OA chondrocytes treated with 0.5, 2, or 4 µg/mL PS aqueous extract compared with that in the untreated group. Meanwhile, the gene expression levels of COL II, ACP, and SOX9 were upregulated by 1.53-, 2.85-, and 1.58-fold, respectively, in human OA chondrocytes treated with 0.5 µg/mL PS aqueous extract compared with those in untreated cells. However, there was no significant difference in the gene expression of COL II, ACP, or SOX9 in human OA chondrocytes treated with 2 or 4 µg/mL PS aqueous extract compared with that in the untreated group.

### 3.5. The Effects of PS Aqueous Extract on Catabolic Gene Expression

Figure 3 shows the gene expression of catabolic mediators (IL-6, MMP1, MMP13, iNOS, and COX2) in human OA chondrocytes treated with different concentrations of PS aqueous extract. COX2 gene expression was significantly downregulated by 0.44- and 0.56-fold, respectively, in OA chondrocytes treated with 2 and 4 µg/mL PS aqueous extract, respectively, compared with that in the untreated cells. However, there was no significant change in the gene expression of IL-6, MMP1, MMP13, or iNOS in human OA chondrocytes treated with any of the three concentrations of the PS aqueous extract.

### 3.6. The Effect of PS Aqueous Extract on NO Production

**Figure 4:**
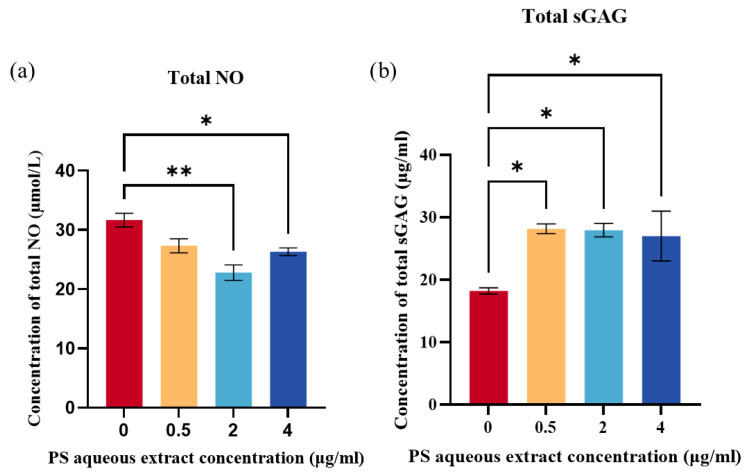
(**a**) Total NO produced by human OA chondrocytes treated with different PS aqueous extract concentrations. (**b**) Total sGAG in human OA chondrocytes treated with different PS aqueous extract concentrations. The values are expressed as the mean ± SEM, N = 3. * *p* < 0.05, ** *p* < 0.01. Nitric oxide, NO; Sulfated glycosaminoglycan, sGAG.

Total NO levels produced by human OA chondrocytes were determined by the analysis of total nitrite and nitrate concentrations. Figure 4a shows the total NO concentration in the human OA chondrocytes after treatment with 0, 0.5, 2, or 4 µg/mL of PS aqueous extract. Following treatment, the concentration of total NO decreased to 31.67 µmol/L, 27.33 µmol/L, 22.8 µmol/L, and 26.33 µmol/L, respectively. The results were significant for chondrocytes treated with 2 and 4 µg/mL PS aqueous extract. This result suggested that the PS aqueous extract can reduce oxidative stress by reducing NO production.

### 3.7. The Effect of PS Aqueous Extract on sGAG Production

The production of sGAG was determined as a measure of ECM production by the human OA chondrocytes. Figure 4b showed that treatment with three different concentrations of PS aqueous extract significantly increased the total sGAG produced by the OA chondrocytes compared with that in untreated cells (*p* < 0.05). The concentrations of total sGAG obtained were as follows: 18.23 µg/mL for the untreated group, 28.17 µg/mL for the 0.5 µg/mL PS aqueous extract group, 27.93 µg/mL for the 2 µg/mL PS aqueous extract group, and 26.99 µg/mL for the 4 µg/mL PS aqueous extract group.

### 3.8. Effect of PS Aqueous Extract on COX2 Protein Expression

**Figure 5:**
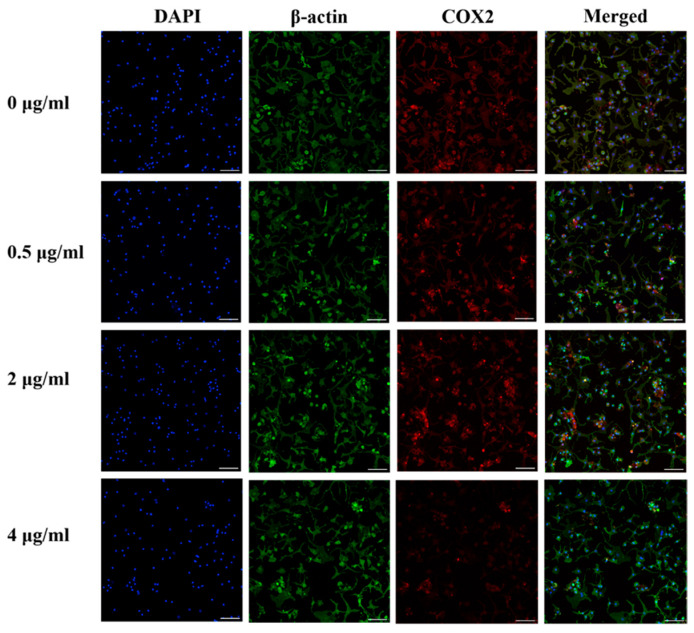
Representative images of immunocytochemical staining for COX2 (red) and β-actin (green) in human OA chondrocytes treated with various PS aqueous extract concentrations at 10× magnification. N = 1. The scale bar represents 100 μm. 4′, 6′-Diamidino-2-phenylindole, DAPI; cyclooxygenase 2, COX2.

Figure 5 shows the results of immunocytochemistry staining of COX2 in human OA chondrocytes. COX2 staining decreased in human OA chondrocytes following treatment with 0.5, 2, or 4 µg/mL of PS aqueous extract, suggesting a reduction in COX2 protein expression. Lowest COX2 protein expression was observed in human OA chondrocytes treated with media supplemented with 4 µg/mL PS aqueous extract.

**Figure 6:**
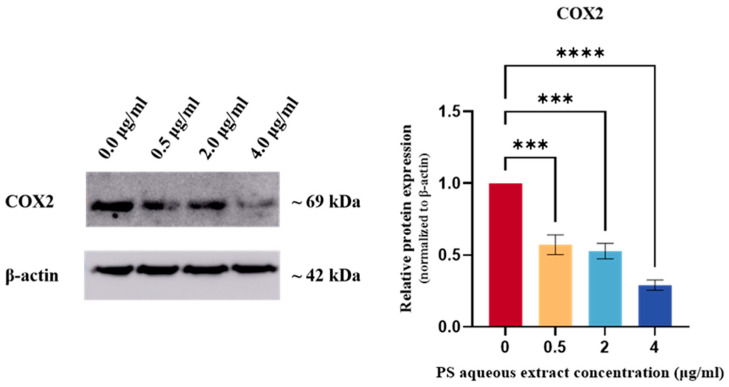
Western blot analysis of COX2 protein expression in human OA chondrocytes treated with different concentrations of PS aqueous extract. The values are expressed as the mean ± SEM, N = 3. *** *p* < 0.001, **** *p* < 0.0001. Cyclooxygenase 2, COX2.

Figure 6 shows the Western blot analysis of COX2 protein expression in human OA chondrocytes. The COX2 protein expression was reduced by 0.57-fold (*p* < 0.001), 0.52-fold (*p* < 0.001), and 0.29-fold (*p* < 0.0001) following treatment with 0.5, 2, and 4 µg/mL of PS aqueous extract, respectively.

### 3.9. Effect of PS Aqueous Extract on the NO Signaling Pathway

**Figure 7:**
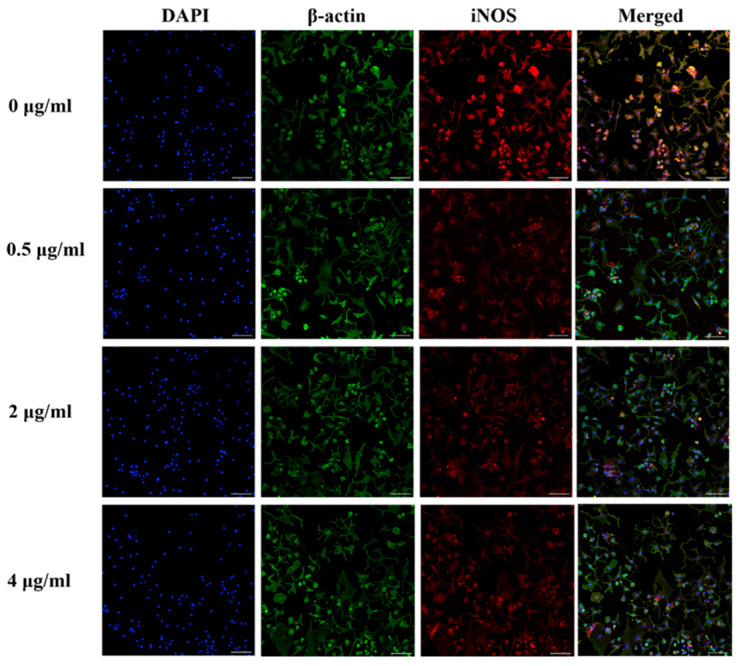
Representative images of immunocytochemical staining of iNOS (red) and β-actin (green) in human OA chondrocytes treated with various concentrations of PS aqueous extract at 10× magnification. The scale bar represents 100 μm. 4′, 6′-Diamidino-2-phenylindole, DAPI; inducible nitric oxide synthase, iNOS.

**Figure 8:**
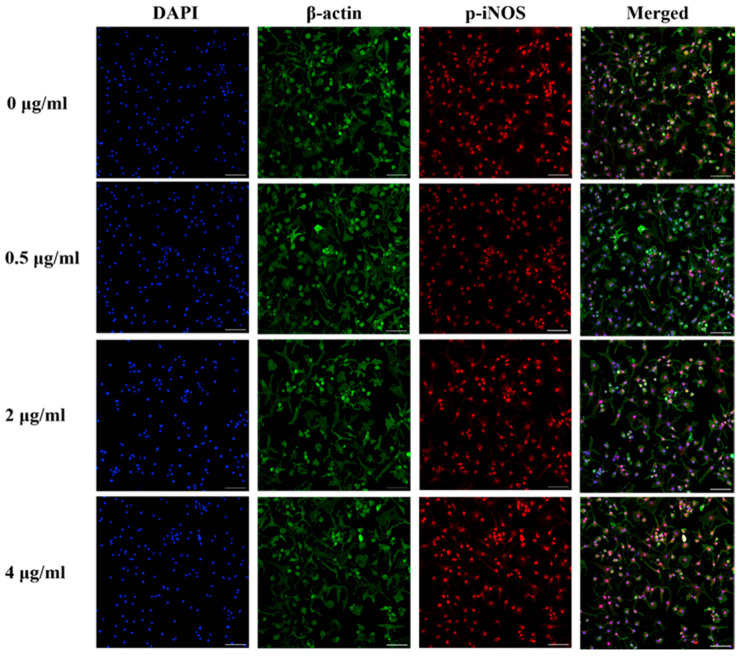
Representative images of immunocytochemical staining of p-iNOS (red) and β-actin (green) in human OA chondrocytes treated with various concentrations of PS aqueous extract at 10× magnification. The scale bar represents 100 μm. 4′, 6′-Diamidino-2-phenylindole, DAPI; phosphorylated-inducible nitric oxide synthase, p-iNOS.

The immunocytochemical staining of iNOS and phosphorylated-iNOS (p-iNOS) in the chondrocytes is shown in Figure 7 and Figure 8. The iNOS protein expression was attenuated in human OA chondrocytes treated with 0.5, 2, and 4 µg/mL PS aqueous extract. However, for the p-INOS protein expression, a slight reduction was observed in human OA chondrocytes treated with 0.5 µg/mL PS aqueous extract, and no difference was observed in the human OA chondrocytes treated with 2 or 4 µg/mL PS aqueous extract compared with those in the untreated group. Additionally, p-iNOS was found in the nucleus of chondrocytes, suggesting that iNOS underwent nuclear translocation during phosphorylation.

**Figure 9:**
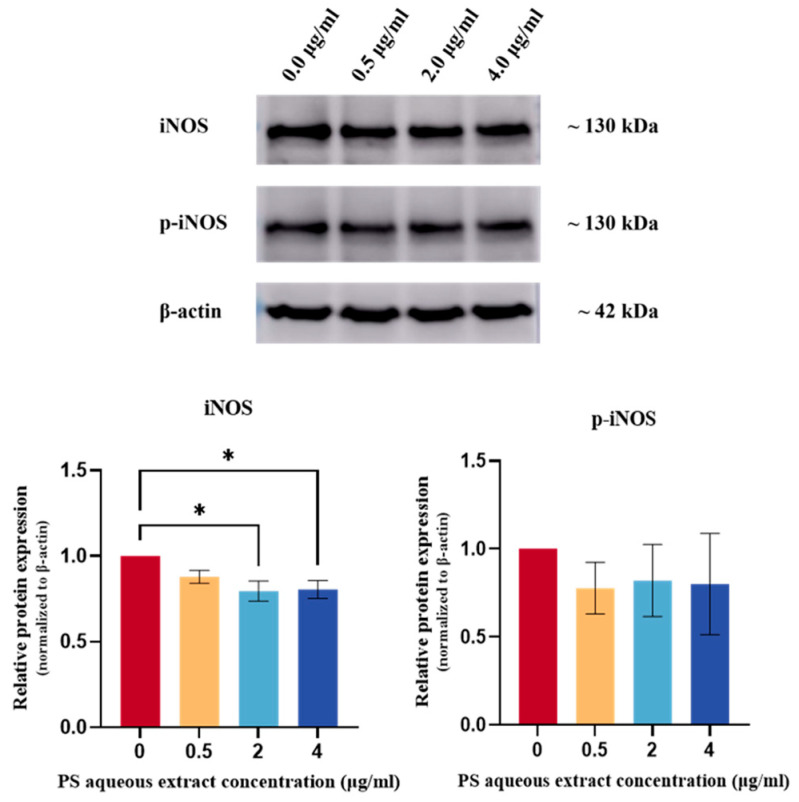
Western blot analysis of iNOS and p-iNOS protein expression in human OA chondrocytes treated with different concentrations of PS aqueous extract. The values are expressed as the mean ± SEM, N = 3. * *p* < 0.05. Inducible nitric oxide synthase, iNOS; phosphorylated-inducible nitric oxide synthase, p-iNOS.

Figure 9 shows the Western blot analysis of iNOS and p-iNOS protein expression in human OA chondrocytes. The iNOS protein expression was reduced by 0.80-fold (*p* < 0.05) and 0.81-fold (*p* < 0.05) in human OA chondrocytes treated with 2 and 4 µg/mL PS aqueous extract, respectively. However, no significant change in p-iNOS protein expression was observed.

## 4. Discussion

Osteoarthritis is a leading cause of disability worldwide, and the prevalence of OA is increasing with increasing life expectancy. The pathophysiology of this disease is complicated and has multiple dynamics [9,42]. With regard to the presence of limited treatments for treating OA, which do not provide satisfactory or long-lasting outcomes, research regarding cartilage repair and regeneration has gained tremendous interest among researchers. There is a pressing need for safer, more effective OA treatments. In this study, the aqueous extract of PS has been used to determine its effectiveness in treating OA. PS is a medicinal plant with various biological activities such as antioxidant, anti-inflammatory, and anti-atherosclerotic properties with no toxic effect [43]. Hence, it might be a safe alternative to stop or reverse OA progression.

The morphology of chondrocytes is closely related to the basic cell function, including cell proliferation, cell spreading, cell differentiation, and inflammation [44]. The actin cytoskeleton in chondrocytes is crucial for arrangement in a cortical ring shape, as the architecture, polymerization status, and connection to the focal adhesion complexes help to determine the phenotype by signaling molecule control [45,46]. Fibroblastic chondrocytes, for instance, enhance COL I and small proteoglycan synthesis, forming a fragile, incompetent ECM known as fibro-cartilaginous tissue [47].This was because the COL II chain consists of higher hydroxylysine, glucosyl, and galactosyl contents, which facilitate proteoglycan linkage [48]. Furthermore, it is important to note that the chondrocytes used in this study were obtained from individuals with severe OA. Notably, prior studies have demonstrated that chondrocytes in OA conditions frequently exhibit a fibroblastic appearance [49].

In this study, a lower PS aqueous extract concentration (0.5 µg/mL) maintained chondrocyte phenotype with minimal proliferation but significantly enhanced the expression of anabolic genes such as COL II, ACP, and SOX9 in chondrocytes. Higher concentrations of PS aqueous extract induced cell proliferation, leading to dedifferentiation and the formation of fibroblastic chondrocytes. Gene expression analysis revealed an increase in COL I gene expression and a decrease in SOX9, COL II, and ACP gene expression in response to 4 µg/mL PS aqueous extract, which is consistent with the previous finding of Mao et al. [50] that chondrocyte dedifferentiation is positively correlated with chondrocyte proliferation. This dedifferentiation correlates with decreased SOX9, COL II, and ACP, and increased COL I expression [51]. However, in this study, there was no significant difference between the 4 µg/mL PS aqueous extract-treated group and the untreated group, suggesting that the chondroprotective properties of PS aqueous extract counteracted the fibroblastic chondrocyte gene expression at high PS aqueous extract concentrations. Furthermore, fibroblastic chondrocytes are normally associated with the elevated cytokine expression [52], providing an explanation for the slight increase in the expression of catabolic genes such as IL-6, MMP1, and MMP13, as the concentration of PS aqueous extract increased. Nonetheless, in this study, chondrocytes treated with PS aqueous extract did not show significant cytokine gene upregulation, suggesting that PS aqueous extract may mitigate cytokine expression induced by fibroblastic chondrocytes.

During OA, the degradation of COL II is irreversible and contributes to the structural and functional integrity of cartilage [53]. Hence, the gene expression of COL II decreases as the disease progresses, with increasing gene expression of COL I, a fibroblastic marker that increases as the disease advances, indicating phenotypic changes in chondrocytes [54,55,56]. Under severe OA conditions, chondrocytes dedifferentiate to form fibroblastic chondrocytes, secrete proteins resembling fibrocartilage, and contribute to stiffness and cartilage with different mechanical properties [54]. In this study, COL I gene expression did not significantly differ between the PS aqueous extract-treated and untreated chondrocytes. In addition, 0.5 µg/mL PS aqueous extract successfully reversed the chondrocyte dedifferentiation by increasing the expression of chondrocyte-specific phenotype markers, including COL II and ACP gene.

SOX9 is a transcription factor that plays a role in chondrogenic cell differentiation and inhibits chondrocyte hypertrophic differentiation [57]. The findings of this study suggest that low concentrations of PS aqueous extract increase SOX9 gene expression, which helps to preserve the chondrocytes’ phenotype. Proteoglycan, which consists of GAG and aggrecan, is integral to cartilage, and GAG provides the backbone for the binding of core proteins to form proteoglycan molecules [58,59]. In this study, the sGAG level increased along with the ACP gene expression when chondrocytes were cultured in 0.5 µg/mL PS aqueous extract, suggesting that PS aqueous extract promoted ECM synthesis.

During OA progression, chondrocytes promote inflammation, triggering MMP synthesis and cartilage degradation [25,60]. PS extract, which is used in traditional medicine for anti-inflammatory purposes, has been utilized to treat various inflammatory conditions and has shown promise in modern medicine by demonstrating anti-inflammatory activity in numerous studies [27,61,62,63,64,65]. Yeo et al. [66] demonstrated that PS extract can alleviate pro-inflammatory cytokine expression in Aβ-induced BV-2 microglial cells. In this study, there were no significant differences in IL-6, MMP1, or MMP13 mRNA expression between the PS aqueous extract-treated group and the untreated group, but augmentation was observed as the concentration of PS increased. This may be attributed to the ability of PS aqueous extract to promote cell proliferation, lead to formation of fibroblastic cartilage and subsequently increasing catabolic gene expression [52]. Despite the significant increase in cell proliferation in the 4 µg/mL PS aqueous extract group, no significant increase in IL-6, MMP1, or MMP13 was observed. This finding suggested that PS aqueous extract does not promote the secretion of cytokines or proteinases involved in cartilage degeneration. Given that IL-6 is a pro-inflammatory cytokine involved in both inflammation and repair, the observed slight increase might indicate a shift towards reparative signaling rather than pro-inflammatory activity [67]. Furthermore, MMP1 and MMP13 are thought to have unique functionality; MMP1 primarily degrades COL I and III, while MMP13 is involved in unwinding collagen triple helices to facilitate further degradation by other MMPs [68,69]. The absence of significant changes in COL I gene expression between the PS aqueous extract-treated group and the untreated group suggested that PS helps prevent OA progression and cartilage fibrosis. Although a slight increase in MMP1 gene expression was observed in this study, this may suggest that PS aqueous extract helps to prevent excessive COL I secretion during OA progression, reducing the risk of fibrosis. A higher PS aqueous extract concentration induces chondrocyte formation with fibroblastic morphology and increases COL I secretion while attempting to limit fibrocartilage formation. However, further research is required to substantiate this statement.

Compared with that in normal cartilage, PGE2 production in cartilage is impulsively increased in OA patients, indicating the upregulation of COX2 transcription, a factor contributing to OA progression. The COX2 protein contributes to PGE2 production, which inhibits ECM synthesis, increases cartilage degradation, and serves as a potential inflammatory mediator [70]. The results of this study demonstrated a significant reduction in COX2 mRNA and protein expression in human OA chondrocytes treated with PS aqueous extract compared to those cultured without supplementation. All the doses showed a reduction in COX2, but 4 µg/mL was a more potent inhibitor due to significant findings from changes in COX2 mRNA and protein expression. The reduction in COX2 suggested that the PS aqueous extract is beneficial for preventing OA progression by reducing the levels of the inflammatory mediator PGE2. Interestingly, the PS aqueous extract significantly reduced COX2 expression while slightly increasing IL-6. This might suggest that the PS aqueous extract contains compounds that inhibit the prostaglandin-mediated pathway, such as pterostilbene [71].

During OA progression, elevated iNOS protein expression leads to the overexpression of NO, a free radical implicated in oxidative stress. NO reacts with oxygen-related free radicals such as hydrogen peroxide, triggering cytotoxic effects and promoting cartilage degradation, fibrosis, and chondrocyte apoptosis [49,72,73]. Moreover, increased NO compromises cell survival by halting the mitochondrial respiratory chain activity and adenosine triphosphate (ATP) synthesis [74]. In this study, treatment with PS aqueous extract at concentrations of 2 and 4 µg/mL significantly reduced iNOS mRNA and protein expression in human OA chondrocytes, accompanied by a notable decrease in NO production. This suggests that PS aqueous extract at concentrations of 2 and 4 µg/mL exhibits antioxidant effects by targeting the NO signaling pathway. Similar results were found in the studies using PS polar extract for the Aβ-induced microglia-mediated neurotoxicity. Yeo et al. [66] suggested that PS aqueous extract reduces NO production through its free radical scavenging activity, a supposition supported by Nguyen et al. [75], who demonstrated that the radical scavenging activity of PS increases with concentration supplementation.

Although the PS aqueous extract significantly reduced iNOS protein expression, it did not significantly reduce p-iNOS levels. This suggested that the PS aqueous extract primarily affects total protein levels rather than targeting the phosphorylation activities in chondrocytes. Phosphorylation regulation is intricate and involves the equilibrium between phosphatase and kinase activities that maintain phosphorylated protein levels [76]. Besides that, phosphorylation of the iNOS protein enhances its stability and prolongs its half-life, explaining the lack of a significant decrease in p-iNOS levels observed in this study [77]. Additionally, phosphorylated proteins often play a role in modulating signal transduction and can affect protein transport into the nucleus [78]. This was evidenced by the presence of p-iNOS in the nucleus of chondrocytes during ICC staining.

The effects of PS aqueous extract are closely related to the compounds present in the extract. Among the 20 most reliable compounds present, 15 of them showed anti-inflammatory, antioxidant, and chondroprotective effects. These compounds include vitexin 4-O-glucoside, palmitic amide, anthranilate, pterostilbene, 3,4,5-trimethoxydihydrocinnamic acid, D-proline, L-asparagine, glutathione, phytosphingosine, L-leucine, dodecanoic acid, tetradecanoic acid, 1,6-anhydro-beta-D-glucose, tryptophan, and 1-palmitoyl-2-(5-keto-8-oxo-6-octenoyl)-sn-glycero-3-phosphocholine. The presence of these bioactive compounds may underlie the molecular effects in our in vitro assays. For example, vitexin 4-O-glucoside, phytosphingosine, and pterostilbene have been reported to exhibit anti-inflammatory and antioxidant effects by reducing the expression of key pro-inflammatory mediators such as TNF-α, IL-1β, IL-6, iNOS, COX2, and PGE2 [71,79,80]. Additionally, glutathione was also detected, which has been shown to play a protective role in osteoarthritis management [81,82]. Compounds such as D-proline and vitexin may also inhibit matrix metalloproteinases (MMPs), thereby helping to prevent cartilage degradation [83]. Moreover, the PS aqueous extract also consists of L-leucine and L-asparagine, which promote protein synthesis and tissue repair [84,85]. Although these represent only a subset of the 101 compounds detected in the extract, their collective presence suggests a potential synergistic effect contributing to the therapeutic outcomes observed in this study.

Overall, this study demonstrated that PS aqueous extract has chondroprotective effects by increasing the synthesis of ECM components (COL II, ACP, SOX9, and GAG) and exerting antioxidant and anti-inflammatory effects on human OA chondrocytes. The significant reductions in COX2 and iNOS protein expression suggest that PS directly targets these pathways. Additionally, the study revealed no significant changes in MMP1 or MMP13 mRNA expression, possibly due to the rapid impact of PS on the NO signaling pathway, which requires more time to affect downstream pathways and proteins [86]. Importantly, the effects of PS aqueous extract were concentration dependent, with lower concentrations favoring anabolic and phenotypic maintenance, supporting the existence of a therapeutic window rather than a linear dose–response relationship. These findings suggest the existence of an optimal therapeutic concentration range, within which PS aqueous extract exerts maximal chondroprotective effects without promoting dedifferentiation. Further studies should explore a more refined concentration range to determine the optimal dose for promoting anabolic activity while avoiding undesired proliferation or dedifferentiation.

This study is a preliminary investigation into the therapeutic potential of PS aqueous extract in mitigating OA progression with several limitations. The results are based on human OA chondrocytes treated with PS aqueous extract for 72 h, but the anti-inflammatory effects of PS are not fully understood due to the dynamic nature of cellular responses. Future studies should collect samples at different time intervals and extend the treatment duration to better understand the underlying mechanism involved. Also, more pathways can be investigated to fully understand the mechanism. Additionally, the use of monolayer cell culture may not accurately mimic the normal cartilage environment, suggesting the need for ex vivo studies to observe chondrocyte behavior in tissues. It should be noted that COL I/COL II protein levels were not measured in this study; therefore, the ratio could not be evaluated, and future studies could include this analysis to better assess chondrocyte phenotypic changes. Also, the findings of this study are specific to basal-state OA chondrocytes. Since normal chondrocytes were not included as controls, it remains unclear whether PS aqueous extract restores the normal phenotype or primarily modulates gene expression in the OA state. Future studies including normal chondrocytes, as well as IL-1β- or TNF-α-stimulated models, are suggested to validate the anti-inflammatory and chondroprotective effects. Lastly, the chemical composition of PS varies depending on its growth environment, affecting its therapeutic capabilities. Therefore, investigating these differences is essential for PS pharmacological and phytochemical research on PS.

## 5. Conclusions

In summary, the results obtained from this study demonstrated that PS aqueous extract could be a potential DMOAD for the treatment of OA. The PS aqueous extract significantly promoted chondroprotection and ECM synthesis, and increased COL II, ACP, SOX9, and GAG production. Furthermore, PS aqueous extract also exerted anti-inflammatory and antioxidant effects on human OA chondrocytes by reducing the synthesis of the COX2 and iNOS proteins and exerted antioxidant effects by targeting the NO signaling pathway. These results suggest that PS aqueous extract might attenuate the severity of OA by reducing inflammation, oxidative stress, and promoting ECM synthesis. Thus, PS aqueous extract is a potential medication for use in OA management.

## Figures and Tables

**Table 1:** The list and sequence of primers used in this study.

Gene Name	Accession Number	Primer Sequence 5′-3′
**GAPDH**	NM_002046	F: 5′-tcc ctg agc tga acg gga ag-3′ R: 5′-gga gga gtg ggt gtc gct gt-3′
**COL I**	NM_000088	F: 5′-agg gct cca acg aga tcg aga-3′ R: 5′-tac agg aag cag aca ggg cca-3′
**COL II**	NM_001844	F: 5′-cta tct gga cga agc agc tgg ca-3′ R: 5′-atg ggt gca atg tca atg atgg-3′
**SOX9**	NM_000346	F: 5′-cac tgt tac cgc cac ttc cc-3′ R: 5′-acc agc gga agt ccc ctt cg-3′
**ACP**	NM_001135	F: 5′-gcg gag gaa gtc ggt gaa ga-3′ R: 5′-ccc tct cgc ttc agg tca gc-3′
**IL6**	NM_000600	F: 5′-tac ccc cag gag aag att cc-3′ R: 5′-ttt tct gcc agt gcc tct tt-3′
**MMP1**	NM_002421	F: 5′-agg tct ctg aag gtc aag ca-3′ R: 5′-ctg gtt gaa aag cat gag ca-3′
**MMP13**	NM_002427	F: 5′-ggt ctt gac cac tcc aag gac-3′ R: 5′-ctc ctc gga gac tgg taa tgg-3′
**INOS**	NM_000625	F: 5′-aca agc cta ccc ctc cag at-3′ R: 5′-tcc cgt cag ttg gta ggt tc-3′
**COX2**	NM_000963	F: 5′-tga gca tct acg gtt tgc tg-3′ R: 5′-tgc ttg tct gga aca act gc-3′

**Table 2:** Top 20 compounds identified based on S/N ratio as an estimate of relative abundance in LC-MS/MS Analysis.

No	Retention Time (min)	Mass (*m*/*z*)	S/N Ratio	Assigned Identification	Adduct	Mass Tolerance (ppm)	Database
**1**	7.8	595.1653	546	Vitexin 4-O-glucoside C_27_H_30_O_15_	[M + H]+	<20	MetaboScape
**2**	9.0	232.1331	374.3	Propyl cinnamate C_12_H_14_O_2_	[M + ACN + H]+	2.05	KEGG
**3**	8.4	274.2738	282.7	Palmitic amide C_16_H_33_NO	[M + H]+	<20	MetaboScape
**4**	1.9	138.0547	189.3	Anthranilate C_7_H_7_NO_2_	[M + H]+	2.04	KEGG
**5**	10.3	274.1436	168	Pterostilbene C_16_H_16_O_3_	[M + NH4]+	0.98	KEGG
**6**	9.1	241.1066	159.8	3,4,5-Trimethoxydihydrocinnamic acid C_12_H_16_O_5_	[M + H]+	<20	MetaboScape
**7**	1.9	116.0701	154	D-Proline C_5_H_9_NO_2_	[M + H]+	<20	MetaboScape
**8**	1.7	133.0604	135.4	L-asparagine C_4_H_8_N_2_O_3_	[M + H]+	<20	MetaboScape
**9**	3.5	174.1488	126.8	gamma-Nonalactone C_9_H_16_O_2_	[M + NH4]+	0.90	KEGG
**10**	1.9	325.1130	117.6	Glutathione C_10_H_17_N_3_O_6_S	[M + NH4]+	15.27	KEGG
**11**	8.5	318.3000	113.2	Phytosphingosine C_18_H_39_NO_3_	[M + H]+	1.42	lipidmaps
**12**	2.8	132.1014	108.8	L-Leucine C_6_H_13_NO_2_	[M + H]+	<20	MetaboScape
**13**	7.2	218.2113	105.5	Dodecanoic acid C_12_H_24_O_2_	[M + NH4]+	1.40	lipidmaps
**14**	3.1	174.1487	101.4	Pelletierine C_8_H_15_NO	[M + CH3OH + H]+	2.34	KEGG
**15**	7.9	246.2424	97.3	Tetradecanoic acid C_14_H_28_O_2_	[M + NH4]+	2.19	lipidmaps
**16**	1.9	163.0602	96.4	1,6-Anhydro-beta-D-glucose C_6_H_10_O_5_	[M + H]+	<20	MetaboScape
**17**	1.7	295.1141	93.5	N-Glycosyl-L-asparagine C_10_H_18_N_2_O_8_	[M + H]+	1.56	KEGG
**18**	8.0	739.2079	89.6	Catheduline E2 C_38_H_40_N_2_O_11_	[M + K]+	26.58	KEGG
**19**	6.34	188.0706	86.6	Tryptophan	M+ H-OH	<20	Plant Metabolites
**20**	7.6	648.3796	84.5	1-Palmitoyl-2-(5-keto-8-oxo-6-octenoyl)-sn-glycero-3-phosphocholine C_32_H_58_NO_10_P	[M + H]+	11.97	KEGG

## Data Availability

The original contributions presented in this study are included in the article/Supplementary Material. Further inquiries can be directed to the corresponding author.

## Supplementary Materials

The following supporting information can be downloaded at https://www.mdpi.com/article/10.3390/biomedicines14010128/s1. Supplementary Materials File S1 is the list of compounds found from the LC-MS/MS results.
