# Supplementary material for: Bioactive-Rich Piper sarmentosum Aqueous Extract Mitigates Osteoarthritic Pathology by Enhancing Anabolic Activity and Attenuating NO-Driven Catabolism in Human Chondrocytes
Source: Biomedicines. 2026 Jan 8;14(1):128. doi: 10.3390/biomedicines14010128 (PMC12838889; doi:10.3390/biomedicines14010128)
Supplement: Supplementary file 1 [file biomedicines-14-00128-s001.zip › biomedicines-4057066-supplementary-File S1.pdf]

INSTITUT BIOLOGI SISTEM
INBIOSIS
INSTITUTE OF SYSTEMS BIOLOGY

CODA®

Analysis Report

For:

Dr. Mohd Heikal Mohd Yunos /

Jabatan Fisiologi
Pusat perubatan Universiti Kebangsaan Malaysia

METHODOLOGY

Analytical Methods

Chromatography

Separation was performed using Thermo Scientific C18 column (AcclaimTM Polar Advantage II, 3 x 150mm, 3um particle size) on Bruker Elute Plus UHPLC, Pump HPG 1300. Gradient elution was performed at flow rate of 0.4ml/min and 40 °C column temperature using H2O + 0.1% Formic Acid (A) and 100% ACN (B) with 22 minutes total run time. The injection volume of sample was 5ul. The gradient started at 5% B (0-3min); 80% B (3-10min); 80% B (10-15min) and 5% B (15- 22min). ii. Mass Spectrometry

Instrument Methods:

Mass spectrometry was carried out using TimsTOF Pro 2 Bruker Daltonic equipped with Electrospray ionisation (ESI) with the following settings:- a) Positive mode ionisation

Acquisition Parameter

Source Type	ESI	Ion Polarity	Positive	Set Nebulizer	2.2 Bar
Scan Begin	20 m/z	Set Capillary	4500 V	Set Dry Heater	220 °C
Scan End	1300 m/z	Set Multipole RF	200.0 Vpp	Set Dry Gas	10.0 l/min
		Set Collision Cell RF	700.0 Vpp	ICC active	Off
		IMS Active	Off	ICC Target	5000000 cts.
		IMS Collision Cell In	140.0 V		

Data Processing

The accurate mass data of the molecular ions, provided by the TOF analyzer, were processed by Compass Data Analysis software (Bruker Daltonik GmbH).

Compound Spectrum List Report

Analysis Info

Analysis	D:\Data\INBIOSIS\2024\Yi Ting\Sample_3_1_342.d	Acquisition Date	2024-10-09 9:19:44 AM
Method	3D-Metabolomics_pos.m	Operator	Admin
Sample	Sample	Instrument	timsTOF Pro 2 1875087.10998
Comment			

Acquisition Parameter

Source Type	ESI	Ion Polarity	Positive	Set Nebulizer	2.2 Bar
Scan Begin	20 m/z	Set Capillary	4500 V	Set Dry Heater	220 °C
Scan End	1300 m/z	Set Multipole RF	200.0 Vpp	Set Dry Gas	10.0 l/min
		Set Collision Cell RF	700.0 Vpp	ICC active	Off
		IMS Active	Off	ICC Target	5000000 cts.
		IMS Collision Cell In	140.0 V		

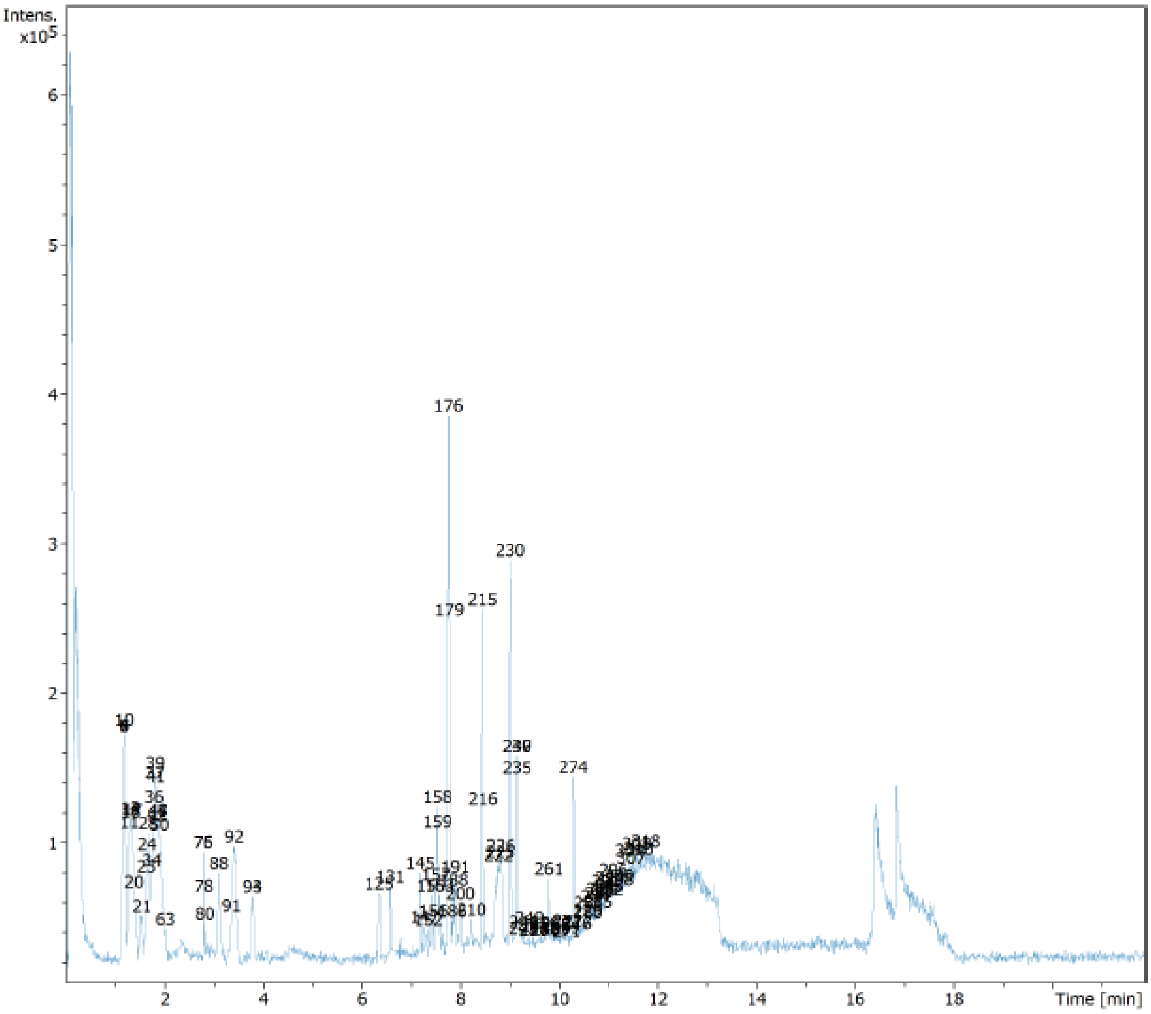

Compound database search paramaters:

- Metaboscape library: Burker NIST 2020 MSMS Spectral Library, Bruker MetaboBase Personal Library 2.0, Bruker HMDB Metabolite Library_2.0, MSDIAL-TandemMassSpectralAtlas-VS69-Pos.msp. , MetFrag.
- Collison energy: 20.0 – 50.0Ev
- Mass Tolerance is below 20ppm unless stated

No	Peak No	Retention time	Mass	S/N	Fragmentation	Assigned identification	Adduct	Mass Tolerance (ppm)	Database
1	4	1.3	110.0081	73.6	39.0233 177<br>42.0341 1668<br>55.9347 777<br>56.9651 831<br>72.9369 219<br>88.0214 108<br>96.0091 239<br>110.0595 260<br>131.9740 401<br>149.9853 108	Unknown			
2	17	1.3	104.1067	59.1	42.0341 685<br>43.0182 226<br>44.0498 2145<br>45.0339 4310<br>45.0576 435<br>58.0655 2193<br>59.0732 545<br>60.0812 5507<br>61.0846 183<br>104.1067 664	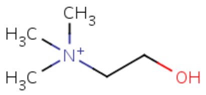<br>Choline<br>$C_5H_{13}NO$	[M+H]+	<20	MetaboScape
3	19	1.34	162.0762	27.6	41.0378 152 8.4<br>42.0348 142 7.9<br>43.0164 158 8.8<br>55.0192 268 14.9<br>60.0446 162 9.0<br>60.0830 142 7.9<br>94.0438 110 6.1	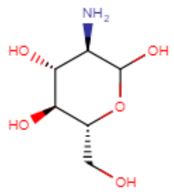<br>D-Glucosamine<br>C6H13NO5	[M+H]+	<20	MetaboScape
4	20	1.4	266.1236	78.8	39.0235 208<br>43.0181 651<br>45.0338 649<br>60.0810 192<br>104.1065 262<br>116.0709 165<br>230.1018 329<br>248.1130 1537<br>266.1584 876<br>267.1630 190	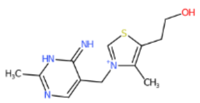<br>Thiamine<br>$C_{12}H_{17}N_4OS$<br>C00378<br><br>Fragments:<br>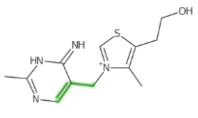<br>39.0235	[M+H]+	14.90	KEGG

No	Peak No	Retention time	Mass	S/N	Fragmentation	Assigned identification	Adduct	Mass Tolerance (ppm)	Database
						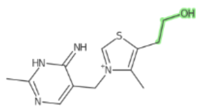<br>45.0338			
5	21	1.5	166.0862	67.7	44.0498 279<br>51.0233 216<br>53.0387 229<br>56.0496 118<br>65.0383 300<br>121.0638 422<br>123.0430 151<br>137.0597 787<br>149.0599 362<br>166.0870 784	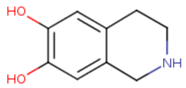<br>1,2,3,4-Tetrahydro-6,7-isoquinolinediol<br>$C_9H_{11}NO_2$	M + H	<20	MetaboScape
6	22	1.6	182.0811	29.3	39.0234 175<br>41.0391 194<br>43.0181 1826<br>45.0337 1788<br>55.9347 311<br>56.9425 901<br>72.9374 250<br>87.0445 272<br>96.0431 330<br>182.0819 148	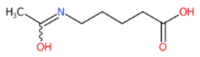<br>5-Acetamidopentanoate<br>$C_7H_{13}NO_3$<br>LMFA01030987<br><br>Fragments:<br>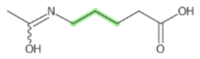<br>41.0391<br>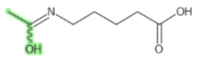<br>43.0181<br>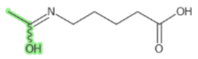<br>45.0337	[M+Na]+	14.39	LipidMaps

No	Peak No	Retention time	Mass	S/N	Fragmentation	Assigned identification	Adduct	Mass Tolerance (ppm)	Database
						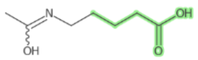<br>87.0445<br>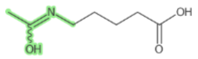<br>96.0431			
7	24	1.65	136.0613	67.9	40.0184 314<br>43.0293 378<br>53.9980 996<br>55.0301 181<br>67.0303 149<br>92.0259 167<br>119.0349 1129<br>136.0609 2318	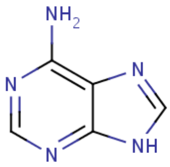<br>Adenine<br>C5H5N5	[M+H]+	<20	MetaboScape
8	28	1.7	133.0604	135.<br>4	43.0181 855<br>44.0134 210<br>44.0498 1318<br>45.0447 187<br>46.0290 720<br>60.0446 111<br>70.0291 249<br>74.0239 1872<br>87.0542 132	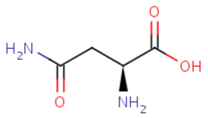<br>L-ASPARAGINE<br>$C_4H_8N_2O_3$	[M+H]+	<20	MetaboScape
9	29	1.7	180.1023	29.0	43.0180 298<br>53.0395 636<br>91.0539 612<br>115.0553 530<br>116.0610 270<br>117.0688 1020<br>145.0632 1292<br>146.0716 352<br>163.0770 1054<br>180.1024 752	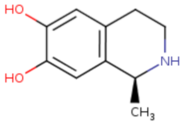<br>Salsolinol<br>$C_{10}H_{13}NO_2$	[M+H]+	<20	MetaboScape
10	32	1.7	457.1666	54.4	45.0342 219<br>122.0576 222<br>150.0574 212<br>194.0429 252<br>211.0703 774<br>241.0826 206<br>259.0946 789<br>277.1002 467<br>421.1458 712<br>439.1569 369	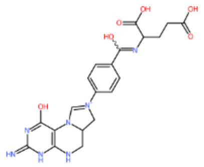<br>5,10-Methenyltetrahydrofolate<br>$C_{20}H_{21}N_7O_6$<br>C00445<br><br>Fragments:	[M+H]+	1.00	KEGG

No	Peak No	Retention time	Mass	S/N	Fragmentation	Assigned identification	Adduct	Mass Tolerance (ppm)	Database
						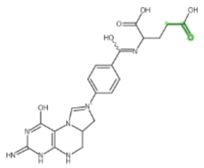<br>45.0342<br>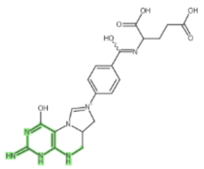<br>122.0576<br>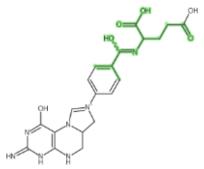<br>194.0429<br>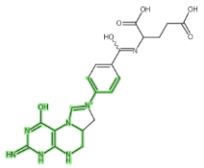<br>241.0826<br>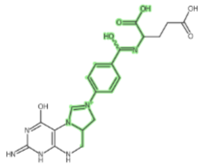<br>259.0946<br>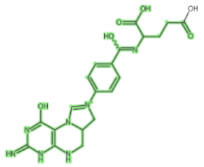<br>439.1569			
11	33	1.7	259.0918	25.2	39.0229 278<br>41.0391 253<br>56.0498 690<br>84.0444 664<br>84.0814 326<br>130.0497 1578<br>213.1256 308	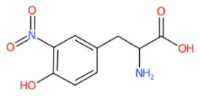<br>3-Nitro-L-tyrosine<br>$C_9H_{10}N_2O_5$<br>C22981<br><br>Fragments:	[M+CH3 OH+H]+	0.58	KEGG

No	Peak No	Retention time	Mass	S/N	Fragmentation	Assigned identification	Adduct	Mass Tolerance (ppm)	Database
						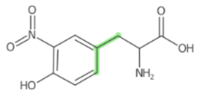<br>39.0229<br>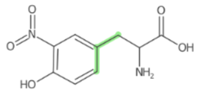<br>41.0391<br>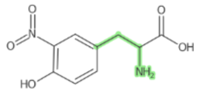<br>56.0498<br>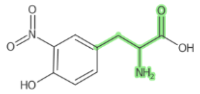<br>84.0444<br>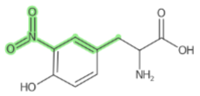<br>130.0497			
12	34	1.7	295.1141	93.5	43.0185 589<br>44.0502 327<br>53.0393 312<br>55.0189 335<br>70.0302 298<br>133.0623 313<br>152.0346 349<br>211.0713 1387<br>259.0937 1438<br>277.1037 392	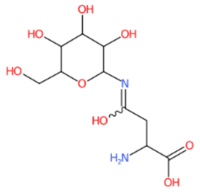<br>N-Glycosyl-L-asparagine<br>$C_{10}H_{18}N_2O_8$<br>C03411<br><br>Fragments:	[M+H]+	1.56	KEGG

No	Peak No	Retention time	Mass	S/N	Fragmentation	Assigned identification	Adduct	Mass Tolerance (ppm)	Database
						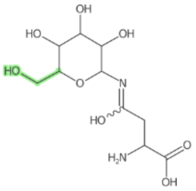<br>43.0185<br>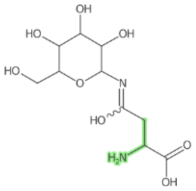<br>44.0502<br>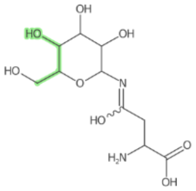<br>55.0189<br>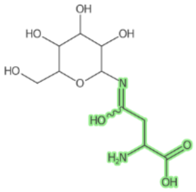<br>133.0623<br>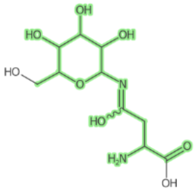<br>259.0937<br>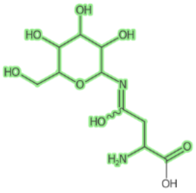<br>277.1037			
13	36	1.6	118.0858	45.0	40.9876 138<br>42.0340 636<br>55.0545 148<br>58.0654 3243<br>59.0733 2639<br>118.0856 552	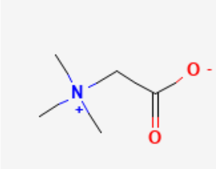<br>BETAINE<br>$C_5H_{11}NO_2$	[M+H]+	<20	MetaboScape

No	Peak No	Retention time	Mass	S/N	Fragmentation	Assigned identification	Adduct	Mass Tolerance (ppm)	Database
14	38	1.8	476.1603	32.8	43.0185 213<br>46.0292 529<br>74.0237 1155<br>88.0395 817<br>116.0337 657<br>127.0382 131<br>133.0612 2156<br>134.0444 4996<br>134.0626 143<br>135.0484 439	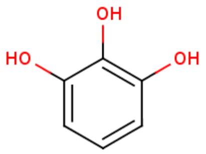<br>Pyrogallol<br>$C_6H_6O_3$<br>Fragments:<br>127.0382 131	[M+H]+	<20	Metaboscape
15	40	1.8	134.0449	37.0	42.0336 185<br>43.0183 3541<br>44.0138 179<br>44.0503 341<br>46.0289 1965<br>70.0290 369<br>74.0244 3215<br>75.0274 129<br>88.0391 589	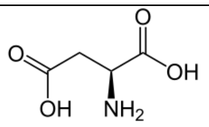<br>L-Aspartic acid<br>$C_4H_7NO_4$	[M+H]+	<20	MetaboScape
16	41	1.9	138.0547	189.3	39.0232 426<br>42.0347 264<br>50.0152 307<br>51.0233 722<br>52.0313 682<br>53.0387 712<br>65.0388 356<br>94.0649 363<br>110.0601 291<br>138.0551 2516	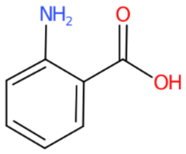<br>Anthranilate<br>$C_7H_7NO_2$<br>C00108<br>Fragments:<br>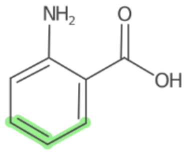<br>39.0232<br>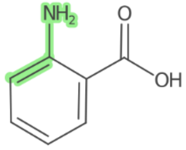<br>42.0347	[M+H]+	2.04	KEGG

No	Peak No	Retention time	Mass	S/N	Fragmentation	Assigned identification	Adduct	Mass Tolerance (ppm)	Database
						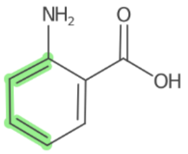<br>50.0152<br>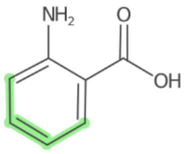<br>51.0233<br>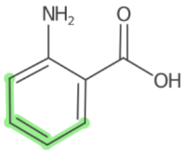<br>52.0313<br>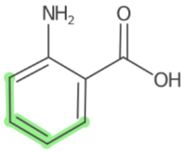<br>53.0387<br>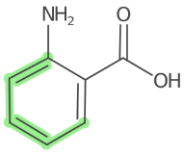<br>65.0388<br>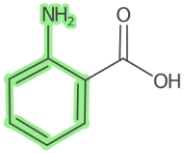<br>94.0649			

No	Peak No	Retention time	Mass	S/N	Fragmentation	Assigned identification	Adduct	Mass Tolerance (ppm)	Database
						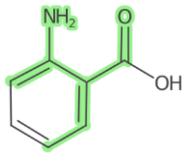<br>110.0601			
17	42	1.8	360.1501	61.8	43.0182 303<br>45.0338 340<br>57.0341 222<br>61.0289 401<br>69.0337 209<br>85.0286 573<br>127.0383 254<br>145.0494 823<br>163.0598 820<br>239.1079 188	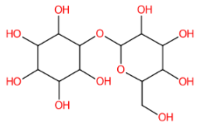<br>alpha-D-Galactosyl-(1->3)-1D-myo-inositol<br>$C_{12}H_{22}O_{11}$<br>C01235<br><br>Fragments:<br>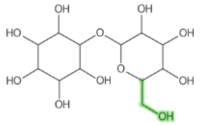<br>43.0182<br>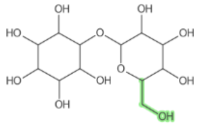<br>45.0338<br>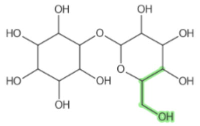<br>57.0341<br>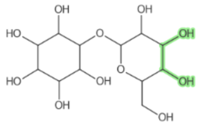<br>61.0289	[M+NH4]+	0.03	KEGG

No	Peak No	Retention time	Mass	S/N	Fragmentation	Assigned identification	Adduct	Mass Tolerance (ppm)	Database
						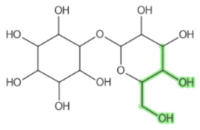<br>85.0286<br>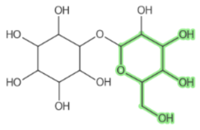<br>145.0494<br>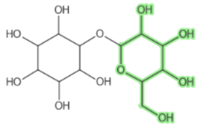<br>163.0598			
18	44	1.9	163.0602	96.4	39.0234 182<br>43.0194 144<br>45.0339 285<br>53.0385 100<br>55.0180 237<br>61.0285 200	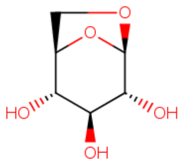<br>1,6-Anhydro-.beta.-D-glucose<br>C6H10O5	[M+H]+	<20	MetaboScape
19	45	2.3	145.0493	22.8	39.0231 252<br>41.0393 120<br>43.0184 318<br>45.0338 177<br>55.0180 266<br>57.0336 209<br>58.0658 134<br>62.9292 152<br>69.0335 220<br>144.1012 264	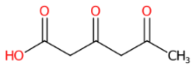<br>Triacetate<br>$C_6H_8O_4$<br>C01757<br><br>Fragments:<br>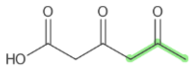<br>39.0231	[M+H]+	1.81	KEGG

No	Peak No	Retention time	Mass	S/N	Fragmentation	Assigned identification	Adduct	Mass Tolerance (ppm)	Database
						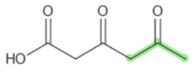<br>41.0393<br>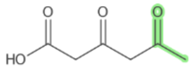<br>43.0184<br>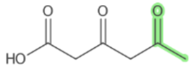<br>45.0338<br>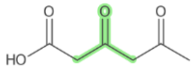<br>55.018<br>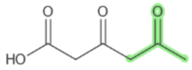<br>57.0336<br>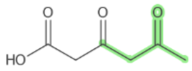<br>69.0335			
20	46	1.9	180.0867	23.6	44.0499 434<br>45.0340 218<br>53.0389 215<br>57.0336 184<br>60.0448 346<br>72.0448 242<br>115.0533 304<br>117.0693 301<br>145.0645 413	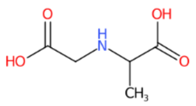<br>N-(Carboxymethyl)-D-alanine	[M+CH3OH+H]+	3.94	KEGG

No	Peak No	Retention time	Mass	S/N	Fragmentation	Assigned identification	Adduct	Mass Tolerance (ppm)	Database
					163.0750 400	$C_5H_9NO_4$<br>C03790<br><br>Fragments:<br>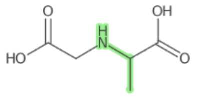<br>44.0499<br>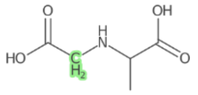<br>45.034<br>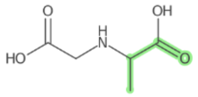<br>57.0336<br>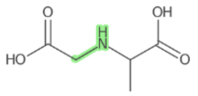<br>60.0448<br>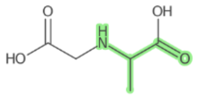<br>72.0448			
21	47	1.9	325.1130	117.6	43.0183 165<br>55.0185 128<br>57.0337 238<br>61.0286 218<br>69.0335 190<br>85.0284 690<br>97.0282 132<br>127.0390 358<br>145.0489 490<br>163.0588 157	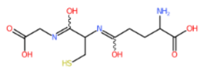<br>Glutathione<br>$C_{10}H_{17}N_3O_6S$<br>C00051<br><br>Fragments:	[M+NH4]+	15.27	KEGG

No	Peak No	Retention time	Mass	S/N	Fragmentation	Assigned identification	Adduct	Mass Tolerance (ppm)	Database
						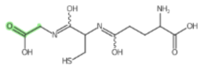<br>43.0183<br>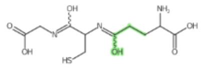<br>55.0185<br>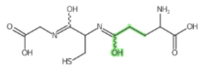<br>57.0337<br>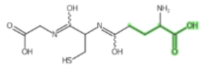<br>85.0284<br>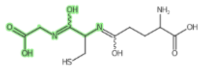<br>145.0489			
22	50	1.9	522.2028	81.0	43.0186 227<br>61.0284 453<br>85.0286 1161<br>109.0274 274<br>127.0387 1233<br>145.0496 2224<br>163.0603 3263<br>289.0930 244<br>325.1123 3086<br>326.1150 376	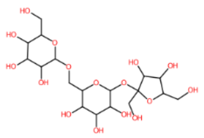<br>Gentianose<br>$C_{18}H_{32}O_{16}$<br>C08239<br>C00492<br><br>Fragments:<br>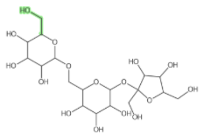<br>43.0186	[M+NH4]+	0.32	KEGG

No	Peak No	Retention time	Mass	S/N	Fragmentation	Assigned identification	Adduct	Mass Tolerance (ppm)	Database
						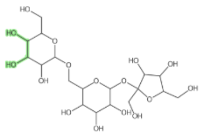<br>61.0284<br>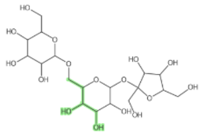<br>85.0286<br>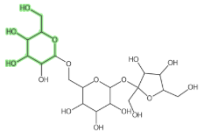<br>145.0496<br>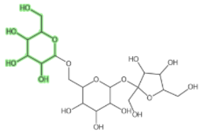<br>163.0603<br>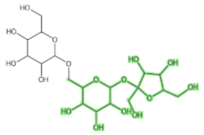<br>325.1123			
23	52	1.9	487.1655	56.8	43.0179 143<br>61.0286 152<br>85.0284 413<br>97.0281 131<br>127.0384 284<br>145.0495 814<br>163.0602 798<br>325.1138 269	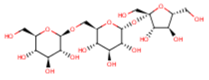<br>Gentianose<br>$C_{18}H_{32}O_{16}$	[M+H]+	<20	MetaboScape
24	54	1.9	130.0859	25.0	39.0232 219<br>41.0387 128<br>42.0339 208<br>55.0544 123<br>55.9350 107<br>56.0498 1382<br>70.0658 858<br>84.0811 1063	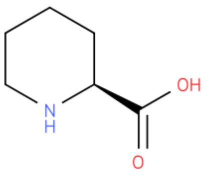<br>L-PIPECOLIC ACID<br>$C_6H_{11}NO_2$	[M+H]+	<20	MetaboScape

No	Peak No	Retention time	Mass	S/N	Fragmentation	Assigned identification	Adduct	Mass Tolerance (ppm)	Database
25	55	1.9	116.0701	154	41.0390 183<br>42.0340 131<br>43.0185 284<br>43.0545 270<br>70.0655 9653<br>71.0683 387<br>116.0692 200	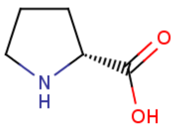<br>D-Proline<br>$C_5H_9NO_2$	[M+H]+	<20	MetaboScape
26	56	2.1	278.1237	32.1	70.0659 444<br>100.0747 156<br>112.0755 174<br>116.0697 236<br>128.0707 459<br>214.1069 226<br>232.1168 340<br>242.1016 744<br>260.1131 1627<br>261.1175 262	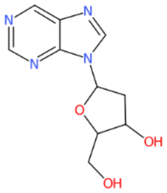<br>Purine deoxyribonucleoside<br>$C_{10}H_{12}N_4O_3$<br>C20463<br><br>Fragments:<br>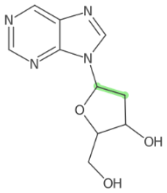<br>70.0659<br>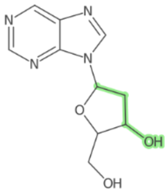<br>100.0747<br>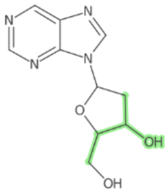<br>112.0755<br>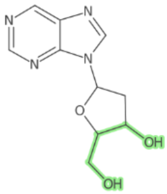<br>116.0697	[M+ACN +H]+	2.29	KEGG

No	Peak No	Retention time	Mass	S/N	Fragmentation	Assigned identification	Adduct	Mass Tolerance (ppm)	Database
						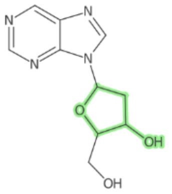<br>128.0707<br>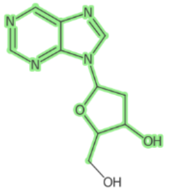<br>260.1131			
27	57	2.0	190.1073	26.4	42.0344 195<br>44.0499 258<br>58.0655 1753<br>59.0734 650<br>60.0811 1239<br>62.9289 154<br>85.0292 174<br>121.9676 214<br>130.0479 102<br>190.1080 421	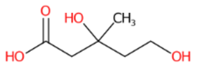<br>(S)-Mevalonate<br>$C_6H_{12}O_4$<br>C02104<br>C00418<br><br>Fragments:<br>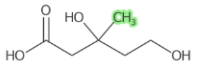<br>58.0655<br>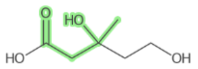<br>85.0292<br>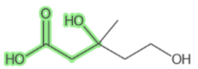<br>130.0479	[M+ACN +H]+	2.90	KEGG

No	Peak No	Retention time	Mass	S/N	Fragmentation	Assigned identification	Adduct	Mass Tolerance (ppm)	Database
28	62	2.0	829.2837	52.6	85.0282 487<br>127.0388 671<br>145.0493 3086<br>163.0602 3194<br>289.0909 244<br>325.1128 4332<br>326.1155 578<br>487.1655 1767<br>488.1683 409<br>649.2200 399	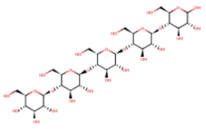<br>Cellopentaose<br>$C_{30}H_{52}O_{26}$	[M+H]+	<20	MetaboScape
29	70	2.7	604.1574	30.7	127.0383 141<br>145.0498 285<br>163.0587 180<br>276.9999 367<br>379.0288 119<br>442.1013 118<br>523.1313 160<br>541.0818 225<br>604.1626 329<br>604.6580 155	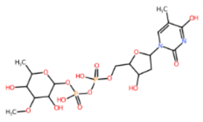<br>dTDP-3-O-methyl-beta-L-rhamnose<br>$C_{17}H_{28}N_2O_{15}P_2$<br>C21350<br><br>Fragments:<br>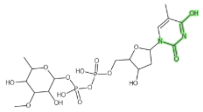<br>127.0383<br>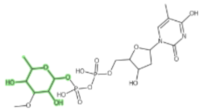<br>145.0498<br>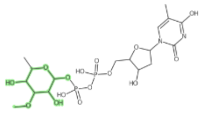<br>163.0587<br>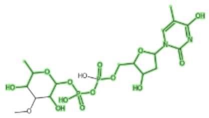<br>541.0818	[M+ACN +H]+	49.07	KEGG

No	Peak No	Retention time	Mass	S/N	Fragmentation	Assigned identification	Adduct	Mass Tolerance (ppm)	Database
30	71	2.7	1153.3967	76.4	127.0384 930<br>145.0490 4785<br>163.0600 9693<br>164.0630 736<br>289.0918 764<br>325.1132 9076<br>326.1160 1204<br>487.1667 2341<br>649.2189 1599<br>811.2730 1133	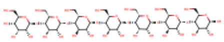<br>Celloheptaose<br>$C_{42}H_{72}O_{36}$	[M+H]+	<20	MetaboScape
31	72	2.8	649.2178	27.9	85.0283 288<br>127.0382 311<br>145.0494 832<br>163.0603 827<br>325.1134 1251<br>326.1164 174<br>487.1647 105	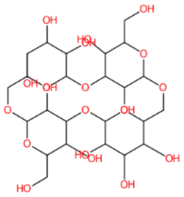<br>Cyclobis-1,6-alpha-nigerosyl<br>$C_{24}H_{40}O_{20}$<br>C21655<br><br>Fragments:<br>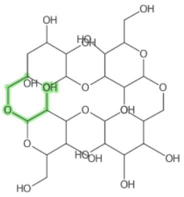<br>85.0283<br>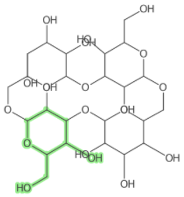<br>127.0382<br>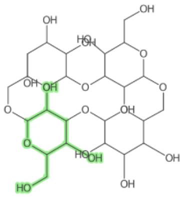<br>145.0494<br>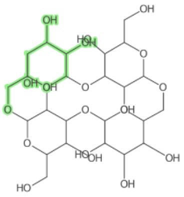<br>163.0603	[M+H]+	1.36	KEGG

No	Peak No	Retention time	Mass	S/N	Fragmentation	Assigned identification	Adduct	Mass Tolerance (ppm)	Database
						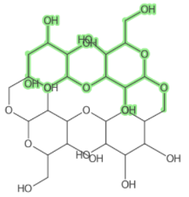<br>325.1134<br>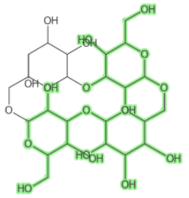<br>487.1647			
32	74	2.8	811.2708	42.9	127.0388 349<br>145.0489 1197<br>163.0598 1058<br>289.0917 102<br>325.1131 1473<br>326.1161 172<br>487.1643 337<br>649.2209 116	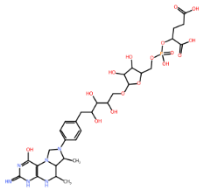<br>5,10-Methylenetetrahydromethanopterin<br>$C_{31}H_{45}N_6O_{16}P$<br>C04377<br><br>Fragments:<br>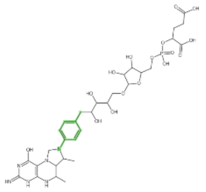<br>127.0388<br>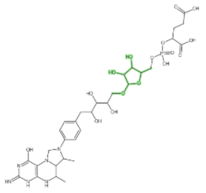<br>145.0489<br>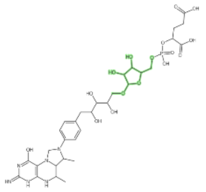<br>163.0598	[M+Na]+	23.46	KEGG

No	Peak No	Retention time	Mass	S/N	Fragmentation	Assigned identification	Adduct	Mass Tolerance (ppm)	Database
						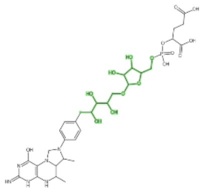<br>289.0917<br>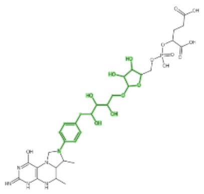<br>325.1131<br>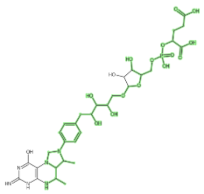<br>649.2209			
33	75	2.8	132.1014	108.8	39.0232 1142<br>41.0387 1002<br>42.0340 312<br>43.0545 771<br>44.0498 3636<br>45.0336 157<br>53.0024 257<br>56.0495 201<br>69.0701 602<br>86.0966 950	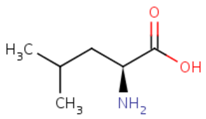<br>L-Leucine<br>$C_6H_{13}NO_2$	[M+H]+	<20	MetaboScape
34	77	2.8	178.0860	27.3	39.0229 329<br>42.0343 307<br>53.0386 222<br>130.0646 218<br>134.0598 250<br>162.0548 844<br>163.0590 209<br>163.0637 297<br>178.0860 3269<br>179.0884 375	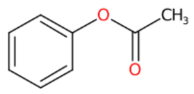<br>Phenyl acetate<br>$C_8H_8O_2$<br>C15583<br>C00548<br><br>Fragments:<br>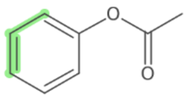<br>39.0229	[M+ACN+H]+	1.98	KEGG

No	Peak No	Retention time	Mass	S/N	Fragmentation	Assigned identification	Adduct	Mass Tolerance (ppm)	Database
						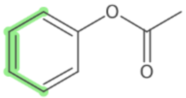<br>53.0386<br>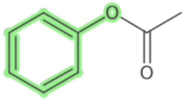<br>134.0598<br>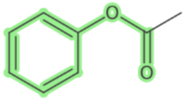<br>162.0548<br>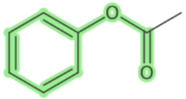<br>163.0637			
35	78	2.8	268.1041	52.7	43.0181 275<br>45.0331 280<br>55.0182 175<br>57.0335 534<br>119.0354 1019<br>136.0616 7246<br>137.0456 192<br>137.0640 347<br>268.1021 281<br>268.1145 123	Tyrosyl-L-leucine	[M+H]+	<20	Plant Metabolites
36	79	2.82	136.0754	24.9	No fragmentation	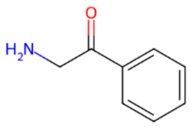<br>2-Amino-1-phenylethan-1-one<br>C8H9NO	[M+H]+	<20	MetaboScape

No	Peak No	Retention time	Mass	S/N	Fragmentation	Assigned identification	Adduct	Mass Tolerance (ppm)	Database
37	80	2.8	182.0814	52.7	39.0230 480<br>45.0337 392<br>51.0236 404<br>53.0389 1371<br>56.9426 433<br>65.0388 816<br>91.0530 555<br>119.0486 836<br>123.0439 1092<br>136.0751 1940	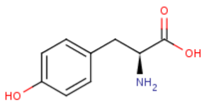<br>L-TYROSINE<br>$C_9H_{11}NO_3$	[M+H]+	<20	MetaboScape
38	82	2.8	294.1548	28.3	41.0390 285<br>42.0346 404<br>44.0498 267<br>45.0339 302<br>86.0959 232<br>132.1012 314<br>144.1017 273<br>230.1385 1056<br>258.1335 1030<br>276.1438 1929	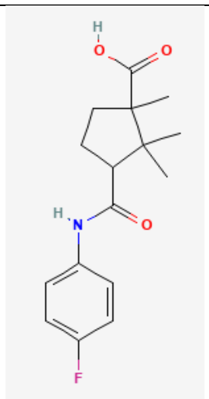<br>3-[(4-Fluoroanilino)carbonyl]-1,2,2-trimethylcyclopentanecarboxylic acid<br>$C_{16}H_{20}FNO_3$	[M+H]+	<20	MetaboScape
39	84	3.1	230.1023	32.9	39.0230 161<br>41.0387 121<br>194.0808 174	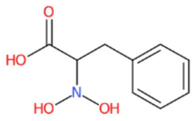<br>N,N-Dihydroxy-L-phenylalanine<br>$C_9H_{11}NO_4$<br>C19715<br><br>Fragments:<br>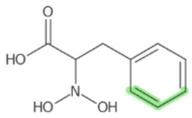<br>39.023	[M+CH3OH+H]+	2.64	KEGG

No	Peak No	Retention time	Mass	S/N	Fragmentation	Assigned identification	Adduct	Mass Tolerance (ppm)	Database
						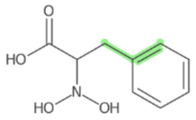<br>41.0387			
40	88	3.1	174.1487	101.4	42.0338 310<br>44.0498 688<br>55.0189 115<br>58.0654 5745<br>59.0734 4143<br>60.0810 1058<br>73.0648 110<br>128.0686 148<br>174.1486 2305<br>175.1521 198	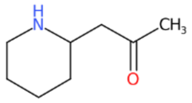<br>Pelletierine<br>$C_8H_{15}NO$<br>C06182<br><br>Fragments:<br>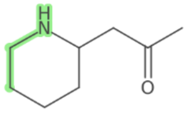<br>42.0338<br>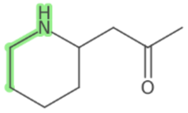<br>44.0498<br>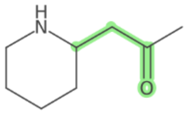<br>55.0189<br>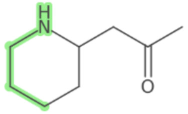<br>58.0654	[M+CH3OH+H]+	2.34	KEGG

No	Peak No	Retention time	Mass	S/N	Fragmentation	Assigned identification	Adduct	Mass Tolerance (ppm)	Database
						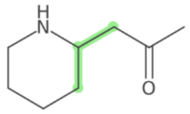<br>73.0648			
41	92	3.5	174.1488	126.8	42.0337 135<br>44.0498 550<br>45.0337 159<br>45.0576 109<br>58.0654 2050<br>59.0733 1279<br>60.0811 893<br>69.0701 116<br>174.1489 788	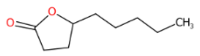<br>gamma-Nonalactone<br>$C_9H_{16}O_2$<br>C08501<br><br>Fragments:<br>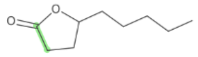<br>42.0337<br>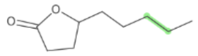<br>44.0498<br>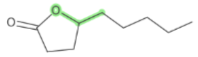<br>45.0337<br>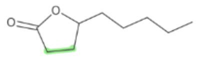<br>45.0576<br>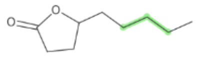<br>58.0654	[M+NH4]+	0.90	KEGG

No	Peak No	Retention time	Mass	S/N	Fragmentation	Assigned identification	Adduct	Mass Tolerance (ppm)	Database
						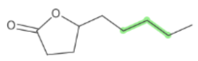<br>59.0733<br>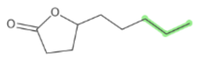<br>60.0811<br>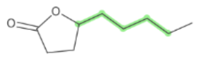<br>69.0701			
42	93	3.8	166.0861	84.3	42.0339 207<br>51.0230 962<br>53.0388 2266<br>65.0390 177<br>77.0399 154<br>79.0539 128<br>91.0534 208<br>103.0531 531<br>120.0803 3632<br>121.0836 360	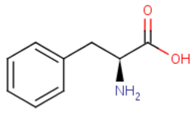<br>L-phenylalanine<br>$C_9H_{11}NO_2$	[M+H]+	<20	MetaboScape
43	95	4.1	98.0601	15.8	39.0231 746<br>41.0388 530<br>42.0341 766<br>44.0134 652<br>53.0026 319<br>53.0389 728<br>55.0181 1065<br>55.9345 607<br>69.0342 678<br>98.0590 307	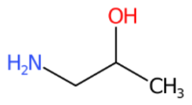<br>1-Aminopropan-2-ol<br>C_3H_9NO<br>C05771<br>C03194<br><br>Fragments:<br>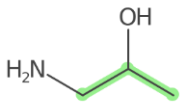<br>41.0388	[M+Na]+	32.37	KEGG

No	Peak No	Retention time	Mass	S/N	Fragmentation	Assigned identification	Adduct	Mass Tolerance (ppm)	Database
						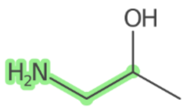<br>42.0341			
44	123	6.3	314.1743	29.8	58.0654 1660<br>269.1158 168<br>314.1735 980<br>315.1778 176	unknown			
45	124	6.3	171.0286	26.4	50.0153 476<br>51.0232 1000<br>53.0389 862<br>55.0180 380<br>81.0347 330<br>107.0126 309<br>109.0279 331<br>125.0239 312<br>127.0380 237<br>153.0190 451	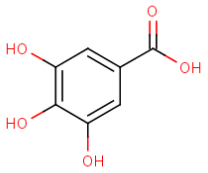<br>Gallic acid $C_7H_6O_5$	[M+H]+	<20	MetaboScape
46	125	6.34	188.0706	86.6	132.0799 480<br>144.0795 907<br>146.0597 2669<br>159.0915 1121<br>170.0591 374<br>188.0705 53328	Tryptophan	M+ H-OH	<20	Plant Metabolites
47	126	6.3	205.0971	41.1	91.0532 531<br>115.0537 1447<br>117.0573 882<br>118.0643 1201<br>130.0646 795<br>142.0649 657<br>143.0717 788<br>144.0807 680<br>146.0598 3652<br>188.0705 1568	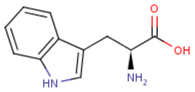<br>L-TRYPTOPHAN $C_{11}H_{12}N_2O_2$	M + H	<20	MetaboScape
48	130	6.4	299.1234	26.9	43.0180 334<br>45.0338 689<br>132.1018 111<br>136.0625 112<br>221.0906 128<br>222.0752 260<br>246.0753 114<br>264.0878 254	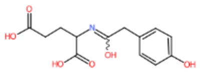<br>4-Hydroxyphenylacetylglutamic acid $C_{13}H_{15}NO_6$ C05595	[M+NH4]+	1.49	KEGG

No	Peak No	Retention time	Mass	S/N	Fragmentation	Assigned identification	Adduct	Mass Tolerance (ppm)	Database
						Fragments:<br>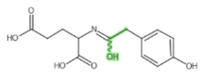<br>43.018<br>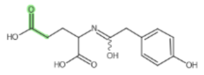<br>45.0338<br>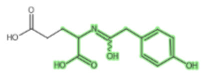<br>222.0752<br>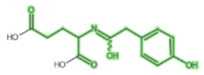<br>246.0753<br>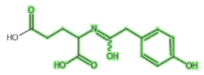<br>264.0878			
49	131	6.6	342.1698	78.2	45.0335 225<br>58.0653 2673<br>165.0686 183<br>191.0865 191<br>222.0684 254<br>265.0852 411<br>297.1116 913<br>298.1170 189<br>342.1698 1365<br>343.1719 442	unknown	[M+H]+	<20	
50	136	6.75	198.1279	17.8	39.0228 320 17.8<br>55.9340 342 19.0<br>60.9734 180 10.0<br>65.0375 1122<br>62.3<br>65.0458 186 10.3<br>71.9303 174 9.7<br>91.0529 1602<br>89.0	3-phenyl acetamide	[M+H]+	<20	Plant Metabolites

No	Peak No	Retention time	Mass	S/N	Fragmentation	Assigned identification	Adduct	Mass Tolerance (ppm)	Database
					96.9963 252 14.0<br>146.9595 206 11.4<br>181.1000 374 20.8				
51	144	7.2	472.2746	27.1	45.0338 2234<br>46.0371 123<br>69.0340 109<br>87.0434 130<br>89.0588 122<br>133.0850 181<br>261.1353 128<br>455.2473 1645<br>456.2509 363<br>472.2704 136	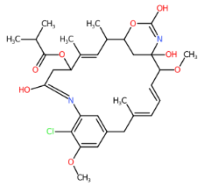<br>N-Demethyl-desepoxyansamitocin P-3<br>$C_{31}H_{41}ClN_2O_8$<br><br>C20137<br><br>Fragments:<br>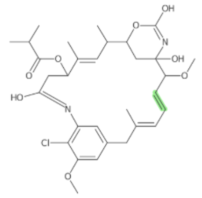<br>44.0494<br>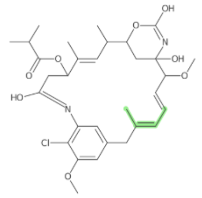<br>70.0657<br>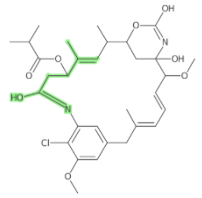<br>129.102	[M+NH4]+	24.59	KEGG

No	Peak No	Retention time	Mass	S/N	Fragmentation	Assigned identification	Adduct	Mass Tolerance (ppm)	Database
						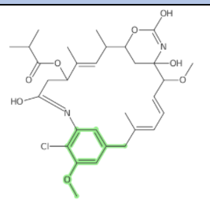<br>136.0766<br>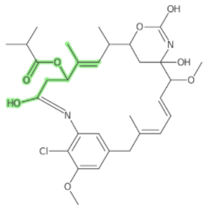<br>159.0906<br>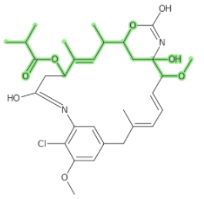<br>301.1901<br>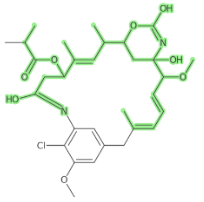<br>434.206<br>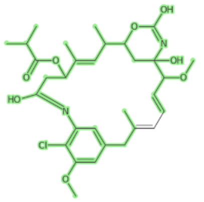<br>610.2895			
52	145	7.2	218.2113	105.5	39.0229 226<br>41.0388 492<br>43.0545 602<br>44.0498 382<br>57.0697 554<br>70.0659 228<br>88.0755 233<br>200.2006 359<br>218.2104 3260<br>219.2130 309	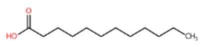<br>Dodecanoic acid<br>$C_{12}H_{24}O_2$<br>LMFA01010012<br><br>Fragments:	[M+NH4]+	1.40	Lipidmaps

No	Peak No	Retention time	Mass	S/N	Fragmentation	Assigned identification	Adduct	Mass Tolerance (ppm)	Database
						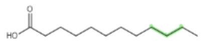<br>41.0388<br>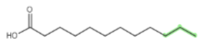<br>43.0545<br>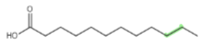<br>44.0498<br>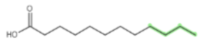<br>57.0697<br>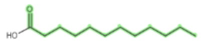<br>200.2006			
53	146	7.2	432.2798	43.0	45.0338 6370<br>89.0590 431<br>133.0856 1010<br>177.1117 601<br>221.1359 131<br>221.1410 151<br>371.2271 153<br>415.2545 1642<br>416.2570 392<br>432.2885 131	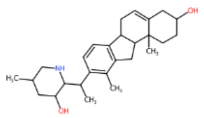<br>Veratramine<br>$C_{27}H_{39}NO_2$<br>C10829<br><br>Fragments:<br>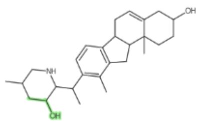<br>45.0338	[M+Na]+	18.76	KEGG

No	Peak No	Retention time	Mass	S/N	Fragmentation	Assigned identification	Adduct	Mass Tolerance (ppm)	Database
						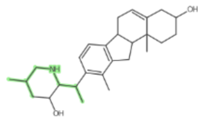<br>133.0856<br>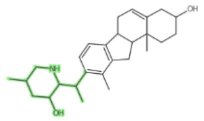<br>177.1117<br>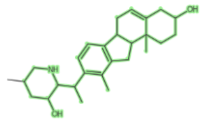<br>416.257			
54	147	7.3	262.2370	51.1	39.0234 361<br>41.0391 515<br>42.0341 454<br>43.0545 701<br>44.0494 383<br>45.0339 539<br>70.0648 327<br>200.2008 1691<br>262.2370 3580<br>263.2416 633	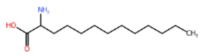<br>2S-Amino-tridecanoic acid<br>$C_{13}H_{27}NO_2$<br>LMFA01100001<br><br>Fragments:<br>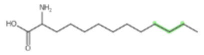<br>41.0391<br>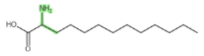<br>42.0341	[M+CH3 OH+H]+	1.05	lipidmaps

No	Peak No	Retention time	Mass	S/N	Fragmentation	Assigned identification	Adduct	Mass Tolerance (ppm)	Database
						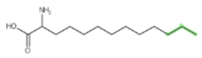<br>43.0545<br>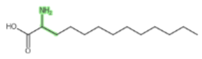<br>44.0494<br>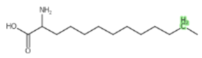<br>45.0339<br>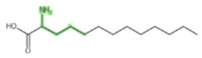<br>70.0648<br>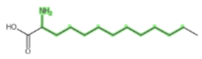<br>200.2008			
55	148	7.3	516.3014	48.9	45.0339 2648<br>69.0345 175<br>89.0589 302<br>133.0841 263<br>305.1655 155<br>499.2737 1872<br>500.2798 533<br>501.2781 151<br>516.3021 632<br>517.3023 184	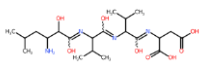<br>Amastatin<br>$C_{21}H_{38}N_4O_8$<br>C01552	[M+ACN +H]+	2.05	KEGG

No	Peak No	Retention time	Mass	S/N	Fragmentation	Assigned identification	Adduct	Mass Tolerance (ppm)	Database
						Fragments:<br>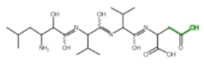<br>45.0339<br>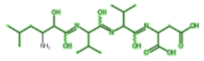<br>499.2737<br>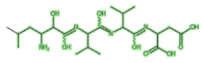<br>501.2781			
56	152	7.8	476.3061	47.7	45.0338 3422<br>122.0808 507<br>133.0840 409<br>177.1087 221<br>177.1151 177<br>221.1336 139<br>459.2783 1083<br>460.2831 173<br>476.3064 197<br>477.3124 111	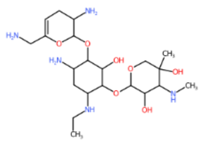<br>Netilmicin<br>$C_{21}H_{41}N_5O_7$<br>C07657<br><br>Fragments:<br>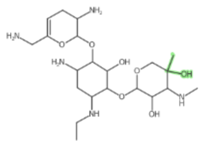<br>45.0338<br>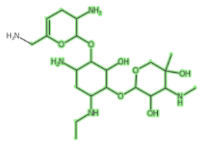<br>459.2783	[M+H]+	4.06	KEGG

No	Peak No	Retention time	Mass	S/N	Fragmentation	Assigned identification	Adduct	Mass Tolerance (ppm)	Database
57	155	7.4	560.3270	84.1	45.0337 2461<br>87.0441 252<br>89.0593 594<br>99.0426 346<br>133.0857 373<br>543.3013 1854<br>544.3048 661<br>560.3277 1407<br>561.3248 275<br>561.3356 253	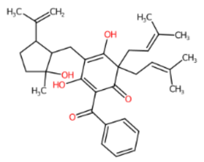<br>Hypercalin B<br>$C_{33}H_{42}O_5$<br>C09941<br><br>Fragments:<br>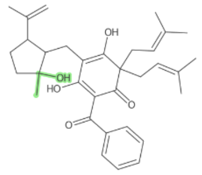<br>45.0337<br>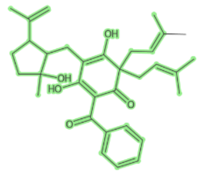<br>544.3048	[M+ACN +H]+	18.68	KEGG
58	156	7.4	520.3321	59.7	45.0338 6567<br>89.0598 321<br>133.0863 654<br>177.1123 469<br>265.1640 301<br>327.1984 147<br>503.3057 2095<br>504.3050 528<br>520.3320 1004<br>521.3316 303	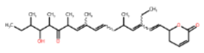<br>Callystatin A<br>$C_{29}H_{44}O_4$<br>C16891<br><br>Fragments<br>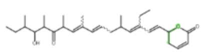<br>45.0338<br>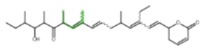<br>133.0863	[M+ACN +Na]+	15.95	KEGG

No	Peak No	Retention time	Mass	S/N	Fragmentation	Assigned identification	Adduct	Mass Tolerance (ppm)	Database
						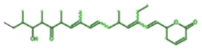<br>504.305			
59	157	7.5	604.3533	83.8	45.0337 1721<br>69.0336 213<br>87.0440 279<br>89.0599 508<br>133.0860 749<br>155.0695 282<br>587.3242 1303<br>588.3314 458<br>604.3570 1586<br>605.3557 455	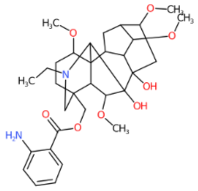<br>Inuline<br>$C_{32}H_{46}N_2O_8$<br>C08659<br><br>Fragments:<br>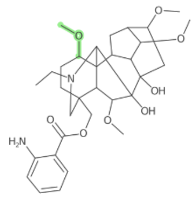<br>45.0337<br>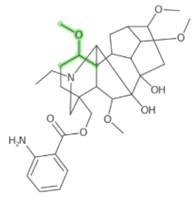<br>69.0336<br>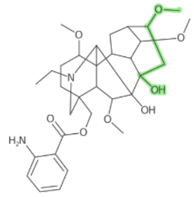<br>87.044<br>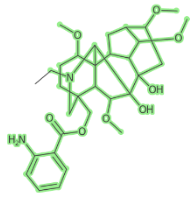<br>588.3314	[M+NH4]+	10.47	KEGG
60	159	7.5	564.3588	69.2	45.0337 7746<br>89.0596 487<br>133.0851 828<br>177.1130 447<br>547.3307 1626<br>548.3364 375<br>564.3583 1639<br>565.2886 139	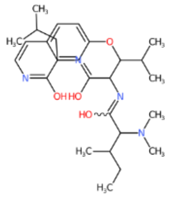<br>Frangulanine	[M+ACN+Na]+	14.27	KEGG

No	Peak No	Retention time	Mass	S/N	Fragmentation	Assigned identification	Adduct	Mass Tolerance (ppm)	Database
					565.3619 597<br>566.3675 214	$C_{28}H_{44}N_4O_4$<br>C10003<br><br>Fragments:<br>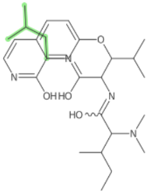<br>133.0851<br>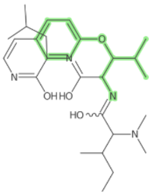<br>177.113			
61	163	7.6	648.3796	84.5	45.0338 3321<br>69.0335 227<br>87.0437 448<br>89.0591 909<br>133.0852 838<br>631.3575 1423<br>632.3506 432<br>648.3798 3035<br>649.3847 790<br>650.3794 273	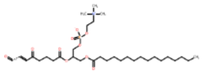<br>1-Palmitoyl-2-(5-keto-8-oxo-6-octenoyl)-sn-glycero-3-phosphocholine<br>$C_{32}H_{58}NO_{10}P$<br>C13902<br><br>Fragments:<br>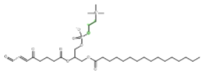<br>45.0338<br>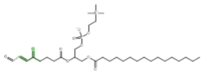<br>69.0335<br>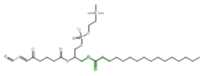<br>87.0437	[M+H]+	11.97	KEGG

No	Peak No	Retention time	Mass	S/N	Fragmentation	Assigned identification	Adduct	Mass Tolerance (ppm)	Database
						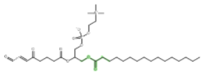<br>89.0591			
62	167	7.6	608.3852	49.8	45.0337 8265<br>46.0370 417<br>89.0590 815<br>133.0860 840<br>177.1114 298<br>591.3580 1695<br>592.3609 467<br>608.3846 3527<br>609.3872 1173<br>611.1594 343	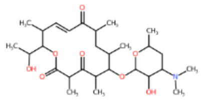<br>C20739<br>Neopikromycin<br>$C_{28}H_{47}NO_8$<br>Fragments:<br>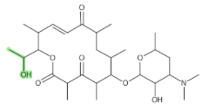<br>45.0337<br>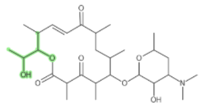<br>89.059<br>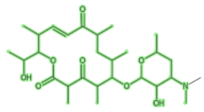<br>592.3609	[M+ACN +H]+	9.48	KEGG
63	168	7.7	692.4059	54.0	45.0338 4201<br>87.0435 266<br>89.0596 661<br>133.0857 793<br>155.0699 361<br>675.3767 1175<br>676.3819 417<br>692.4032 3644<br>693.4062 1428<br>694.4070 451	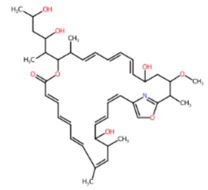<br>Chivosazole F<br>$C_{41}H_{57}NO_8$<br>C15733<br><br>Fragments:	[M+H]+	14.52	KEGG

No	Peak No	Retention time	Mass	S/N	Fragmentation	Assigned identification	Adduct	Mass Tolerance (ppm)	Database
						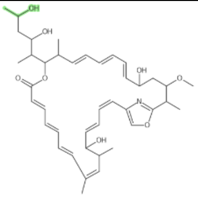<br>45.0338<br>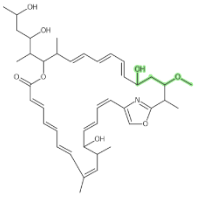<br>87.0435<br>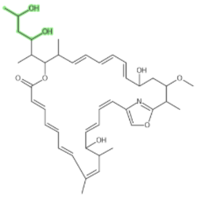<br>89.0596<br>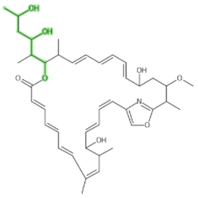<br>133.0857<br>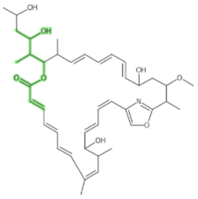<br>155.0699<br>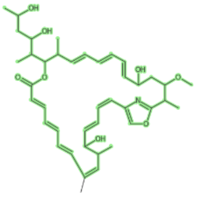<br>675.3767<br>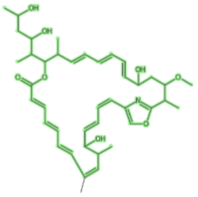<br>676.3819			

No	Peak No	Retention time	Mass	S/N	Fragmentation	Assigned identification	Adduct	Mass Tolerance (ppm)	Database
64	171	7.7	652.4108	29.6	45.0337 5449<br>89.0596 821<br>133.0851 958<br>221.1370 211<br>635.3866 1183<br>636.3850 478<br>652.4135 2867<br>653.3354 195<br>653.4162 1169<br>654.4210 169	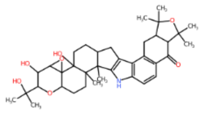<br>Lolitriol<br>$C_{37}H_{49}NO_7$<br>C20548<br><br>Fragments:<br>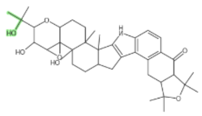<br>45.0337<br>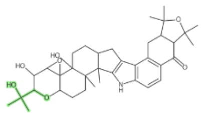<br>89.0596<br>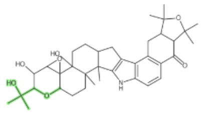<br>133.0851<br>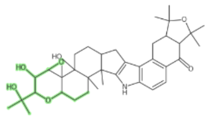<br>221.137<br>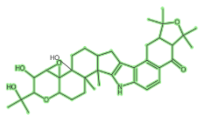<br>636.385	[M+CH3 OH+H]+	43.19	KEGG

No	Peak No	Retention time	Mass	S/N	Fragmentation	Assigned identification	Adduct	Mass Tolerance (ppm)	Database
65	172	7.7	736.4325	42.0	45.0338 2861<br>89.0590 684<br>133.0854 657<br>155.0701 203<br>177.1141 219<br>719.4038 772<br>720.4145 285<br>736.4313 2996<br>737.4405 1166<br>738.4450 345	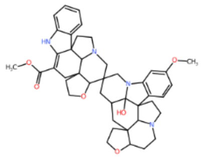<br>Vobtusine<br>$C_{43}H_{50}N_4O_6$<br>C09254<br><br>Fragments:<br>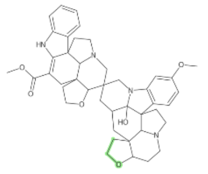<br>45.0338<br>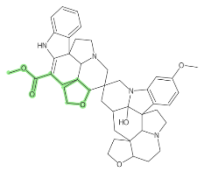<br>155.0701<br>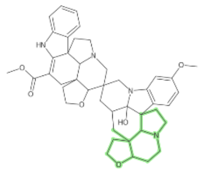<br>177.1141<br>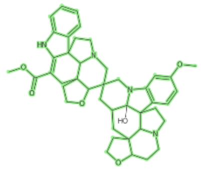<br>719.4038<br>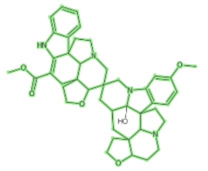<br>720.4145	[M+NH4]+	35.39	KEGG

No	Peak No	Retention time	Mass	S/N	Fragmentation	Assigned identification	Adduct	Mass Tolerance (ppm)	Database
66	174	7.7	377.2328	33.4	45.0337 13539<br>46.0369 359<br>69.0335 966<br>89.0597 467<br>133.0857 902<br>163.0377 311<br>177.1124 412<br>197.1097 269<br>241.1345 333<br>261.1333 249	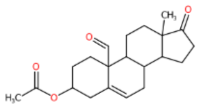<br>3beta-Hydroxy-17-oxoandrost-5-en-19-al acetate<br>$C_{21}H_{28}O_4$<br>C15135<br><br>Fragments:<br>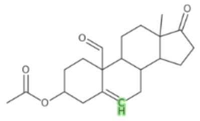<br>45.0337<br>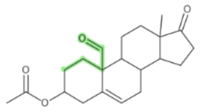<br>69.0335<br>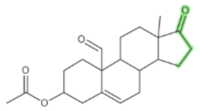<br>89.0597<br>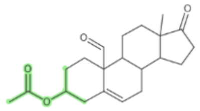<br>133.0857<br>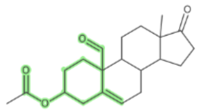<br>163.0377	[M+CH3OH+H]+	2.85	KEGG

No	Peak No	Retention time	Mass	S/N	Fragmentation	Assigned identification	Adduct	Mass Tolerance (ppm)	Database
67	176	7.8	595.1653	546	271.0606 2124<br>283.0599 3324<br>295.0599 1802<br>313.0705 14556<br>314.0738 2472<br>337.0700 2310<br>397.0911 3152<br>415.1028 2437<br>433.1127 3544<br>595.1661 5976	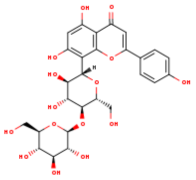<br>Vitexin 4-O-glucoside<br>$C_{27}H_{30}O_{15}$	[M+H]+	<20	MetaboScape
68	179	7.8	399.2457	57.2	43.0176 315<br>45.0338 12154<br>59.0486 285<br>69.0340 731<br>89.0600 713<br>133.0855 1134<br>177.1125 461<br>219.1229 397<br>241.1368 370<br>263.1517 423	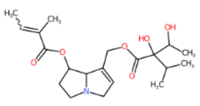<br>Symphytine<br>$C_{20}H_{31}NO_6$<br>C10409<br>C10408<br><br>Fragments;<br>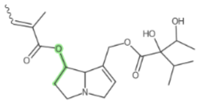<br>43.0176<br>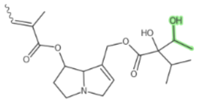<br>45.0338<br>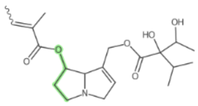<br>59.0486<br>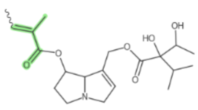<br>69.034	[M+NH4]+	8.92	KEGG

No	Peak No	Retention time	Mass	S/N	Fragmentation	Assigned identification	Adduct	Mass Tolerance (ppm)	Database
						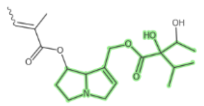<br>263.1517			
69	180	7.8	390.7319	38.4	43.0184 114<br>45.0337 4265<br>46.0369 116<br>55.0536 127<br>69.0336 353<br>73.0289 160<br>89.0591 174<br>133.0858 285<br>177.1118 214<br>177.1645 140	unknown			
70	181	7.8	544.2953	32.5	45.0338 3423<br>69.0339 325<br>73.0284 705<br>289.1293 211<br>499.2754 774<br>527.2691 1971<br>528.2676 392<br>528.2859 190<br>544.2998 1055<br>545.2984 317	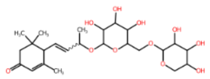<br>C17888<br>Eriojaposide A<br>$C_{24}H_{38}O_{11}$<br><br>Fragments:<br>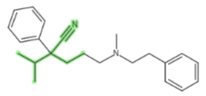<br>133.0856<br>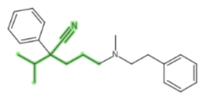<br>146.0945	[M+ACN +H]+	40.78	KEGG
71	185	7.8	412.7456	32.9	45.0337 4879<br>46.0374 178<br>69.0337 399<br>73.0289 291<br>87.0444 150<br>89.0595 170<br>133.0855 324<br>177.1090 184<br>219.1248 188<br>241.1338 137	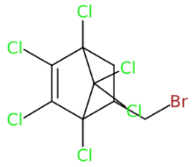<br>Bromocyclen<br>$C_8H_5BrCl_6$<br>C18958	[M+Na] +	36.46	KEGG

No	Peak No	Retention time	Mass	S/N	Fragmentation	Assigned identification	Adduct	Mass Tolerance (ppm)	Database
						Fragments:<br>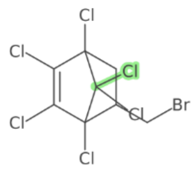<br>45.0337<br>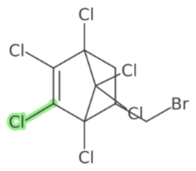<br>46.0374<br>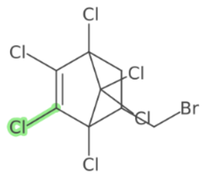<br>69.0337			
72	186	7.8	421.2597	80.8	45.0337 8477<br>55.0543 255<br>69.0333 538<br>89.0595 615<br>111.0436 327<br>133.0855 872<br>155.0699 217<br>177.1104 388<br>263.1442 193<br>404.2337 487	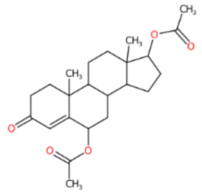<br>6beta,17beta-Dihydroxyandrost-4-en-3-one diacetate<br>$C_{23}H_{32}O_5$<br>C15284<br><br>Fragments:<br>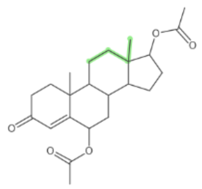<br>55.0543<br>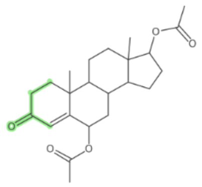<br>69.0333	[M+CH3OH+H]+	4.25	KEGG

No	Peak No	Retention time	Mass	S/N	Fragmentation	Assigned identification	Adduct	Mass Tolerance (ppm)	Database
						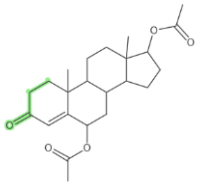<br>89.0595<br>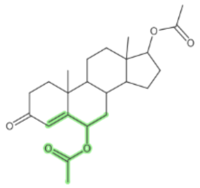<br>111.0436<br>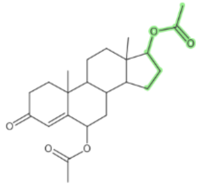<br>133.0855<br>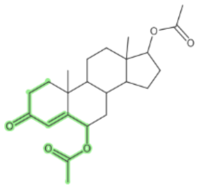<br>155.0699			
73	187	7.8	504.3000	23.9	45.0340 3842<br>73.0292 1311<br>89.0592 254<br>133.0850 264<br>177.1110 257<br>459.2773 617<br>487.2727 1006<br>488.2760 271<br>504.2992 726<br>505.3042 214	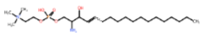<br>Sphingosyl-phosphocholine<br>$C_{23}H_{49}N_2O_5P$<br>LMSP01060001<br>Fragments:<br>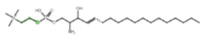	[M+K]+	0.99	Lipidmaps

No	Peak No	Retention time	Mass	S/N	Fragmentation	Assigned identification	Adduct	Mass Tolerance (ppm)	Database
						45.034<br>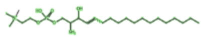<br>488.276			
74	188	7.9	579.1708	81.6	283.0589 1175<br>313.0718 4132<br>314.0750 813<br>337.0657 627<br>343.0801 687<br>397.0912 1753<br>415.1028 1093<br>433.1122 2660<br>579.1709 3821<br>580.1723 981	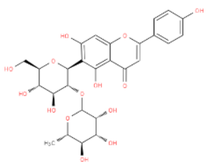<br>Vitexin-2''-O-rhamnoside<br>$C_{27}H_{30}O_{14}$	[M+H]+	<20	MetaboScape
75	189	7.9	588.3223	50.7	45.0337 2985<br>69.0344 229<br>73.0287 748<br>89.0587 500<br>133.0851 284<br>543.3014 367<br>571.2964 1238<br>572.3023 432<br>588.3221 1977<br>589.3234 413	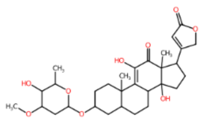<br>Decoside<br>$C_{30}H_{42}O_{9}$<br>C08860<br><br>Fragments:<br>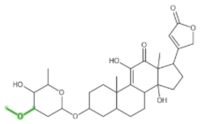<br>45.0337<br>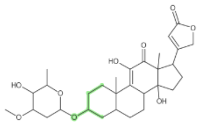<br>69.0344<br>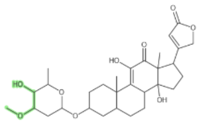<br>73.0287	[M+ACN+H]+	10.95	KEGG

No	Peak No	Retention time	Mass	S/N	Fragmentation	Assigned identification	Adduct	Mass Tolerance (ppm)	Database
						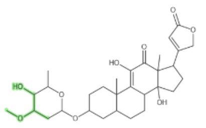<br>89.0587<br>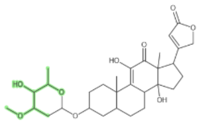<br>133.0851			
76	190	7.9	443.2722	33.8	45.0337 8307<br>69.0335 435<br>89.0599 516<br>133.0853 1153<br>147.0440 199<br>177.1113 394<br>199.0957 222<br>241.1325 220<br>263.1484 204<br>426.2469 262	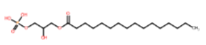<br>1-Palmitoylglycerol 3-phosphate<br>$C_{19}H_{39}O_7P$<br><br>LMGP10050006 LMGP10050041<br><br>Fragments:<br>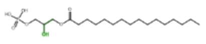<br>45.0337<br>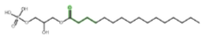<br>69.0335<br>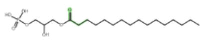<br>89.0599	[M+CH3OH+H]+	10.34	Lipidmaps

No	Peak No	Retention time	Mass	S/N	Fragmentation	Assigned identification	Adduct	Mass Tolerance (ppm)	Database
						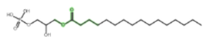<br>33.0853<br>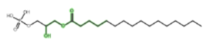<br>177.1113			
77	191	7.9	246.2424	97.3	39.0234 470<br>41.0390 907<br>42.0338 240<br>43.0545 521<br>44.0501 670<br>45.0339 395<br>57.0699 514<br>228.2319 529<br>246.2432 4621<br>247.2471 838	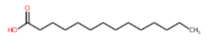<br>Tetradecanoic acid<br>$C_{14}H_{28}O_2$<br>LMFA01010014<br>Fragments:<br>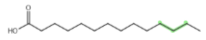<br>41.039<br>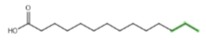<br>43.0545<br>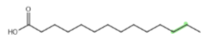<br>44.0501	[M+NH4]+	2.19	Lipidmaps

No	Peak No	Retention time	Mass	S/N	Fragmentation	Assigned identification	Adduct	Mass Tolerance (ppm)	Database
						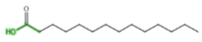<br>45.0339<br>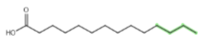<br>57.0699<br>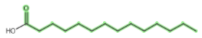<br>228.2319			
78	192	7.9	548.3267	31.5	45.0337 3231<br>73.0286 706<br>133.0852 333<br>177.1131 220<br>205.1080 321<br>503.3090 276<br>531.2987 895<br>532.2998 295<br>548.3265 1253<br>549.3326 488	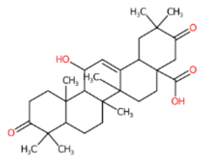<br>Propapyriogenin A2<br>$C_{30}H_{44}O_5$<br>C08971<br><br>Fragments:<br>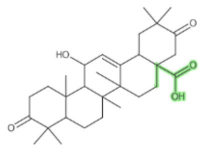<br>73.0286<br>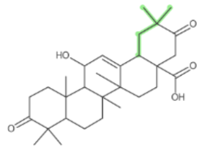<br>133.0852	[M+ACN+Na]+	15.67	KEGG

No	Peak No	Retention time	Mass	S/N	Fragmentation	Assigned identification	Adduct	Mass Tolerance (ppm)	Database
						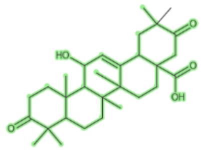<br>532.2998			
79	193	7.9	465.2847	24.0	45.0338 2987<br>46.0369 184<br>69.0332 191<br>87.0445 159<br>89.0587 152<br>122.0812 404<br>177.1110 145<br>241.1360 125<br>263.1481 149<br>285.1612 164	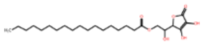<br>L-Ascorbic acid, 6-octadecanoate<br>$C_{24}H_{42}O_7$<br>C20340<br><br>Fragments:<br>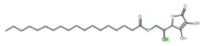<br>45.0338<br>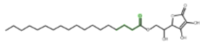<br>69.0332<br>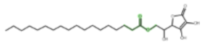<br>87.0445	[M+Na]+	5.09	KEGG
80	194	8.3	290.2688	40.6	39.0231 242<br>41.0387 369<br>43.0544 433<br>44.0498 296<br>45.0341 407<br>57.0701 370<br>70.0650 246<br>228.2324 739<br>290.2685 3126<br>291.2720 601	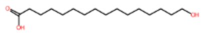<br>16-Hydroxypalmitate<br>$C_{16}H_{32}O_3$<br>LMFA01050051<br><br>Fragments:	[M+NH4]+	1.21	Lipidmaps

No	Peak No	Retention time	Mass	S/N	Fragmentation	Assigned identification	Adduct	Mass Tolerance (ppm)	Database
						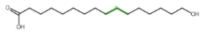<br>41.0387<br>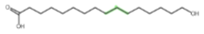<br>43.0544<br>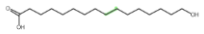<br>44.0498<br>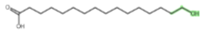<br>45.0341<br>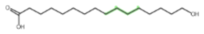<br>57.0701<br>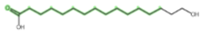<br>228.2324			
81	195	7.9	632.3482	53.2	45.0339 3010<br>69.0337 252<br>73.0285 873<br>87.0437 227<br>89.0589 315<br>133.0847 317<br>615.3235 1217<br>616.3267 493<br>632.3490 2604<br>633.3490 980	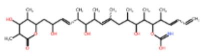<br>Discodermolide<br>$C_{33}H_{55}NO_8$<br>C16746<br><br>Fragments:	[M+K]+	13.41	KEGG

No	Peak No	Retention time	Mass	S/N	Fragmentation	Assigned identification	Adduct	Mass Tolerance (ppm)	Database
						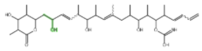<br>45.0339<br>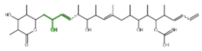<br>69.0337<br>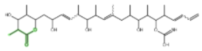<br>73.0285<br>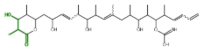<br>87.0437<br>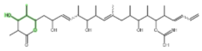<br>89.0589<br>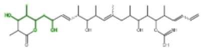<br>133.0847			
82	199	8.0	592.3526	36.4	45.0337 4623<br>73.0290 985<br>89.0593 457<br>133.0853 341<br>205.1060 303<br>221.1381 185<br>575.3227 1057<br>576.3270 305<br>592.3533 2256<br>593.3541 533	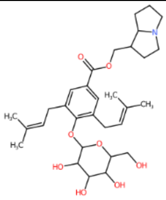<br>Auriculine<br>$C_{31}H_{45}NO_8$<br>C10280	[M+CH3 OH+H]+	8.87	KEGG

No	Peak No	Retention time	Mass	S/N	Fragmentation	Assigned identification	Adduct	Mass Tolerance (ppm)	Database
						Fragments:<br>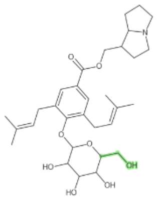<br>45.0337<br>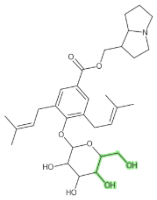<br>73.029<br>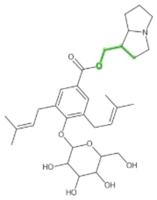<br>89.0593<br>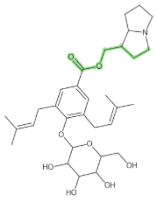<br>133.0853			
83	200	8.0	739.2079	89.6	271.0591 714<br>295.0589 626<br>313.0700 6103<br>314.0729 853<br>337.0696 516<br>415.1032 459<br>475.1257 678<br>577.1533 512<br>739.2071 1887<br>740.2136 803	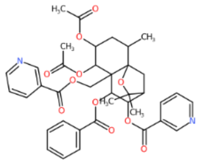<br>Catheduline E2<br>$C_{38}H_{40}N_{2}O_{11}$<br>C09935<br>Fragments:<br>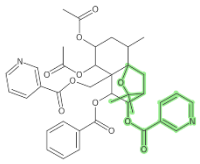<br>271.0591	[M+K]+	26.58	KEGG

No	Peak No	Retention time	Mass	S/N	Fragmentation	Assigned identification	Adduct	Mass Tolerance (ppm)	Database
						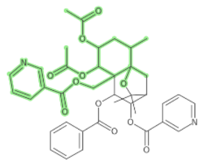<br>415.1032<br>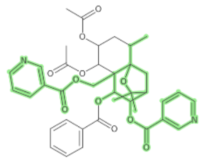<br>475.1257			
84	202	8.0	676.3742	46.6	45.0338 3028<br>73.0283 987<br>133.0853 332<br>155.0692 241<br>631.3574 285<br>659.3526 1009<br>660.3515 239<br>676.3738 3331<br>677.3783 992<br>678.3782 241	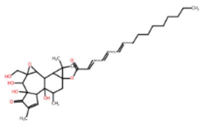<br>Mancinellin<br>$C_{36}H_{52}O_8$<br>C09127<br><br>Fragments:<br>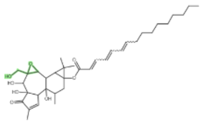<br>73.0283<br>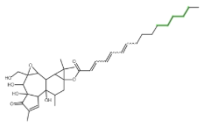<br>133.0853<br>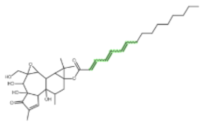<br>155.0692	[M+ACN+Na]+	12.18	KEGG

No	Peak No	Retention time	Mass	S/N	Fragmentation	Assigned identification	Adduct	Mass Tolerance (ppm)	Database
						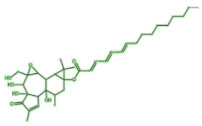 660.3515			
85	205	8.1	720.4005	38.0	45.0337 2187<br>69.0338 237<br>73.0287 539<br>89.0608 224<br>133.0848 445<br>703.3708 369<br>703.3857 359<br>720.4028 2271<br>721.4085 874<br>722.4106 346	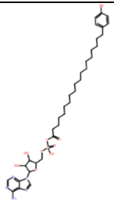 19-(4-Hydroxyphenyl)nonadecanoyl adenylate $C_{35}H_{54}N_5O_9P$<br><br>C21447<br><br>Fragments:<br>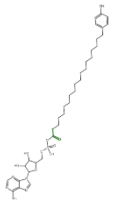 45.0337<br>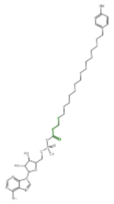 69.0338<br>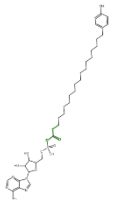 73.0287<br>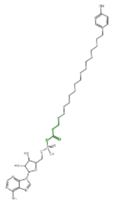 89.0608	[M+H]+	37.67	KEGG

No	Peak No	Retention time	Mass	S/N	Fragmentation	Assigned identification	Adduct	Mass Tolerance (ppm)	Database
						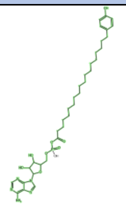 703.3708			
86	206	8.1	433.1127	47.7	283.0598 1545<br>284.0672 1568<br>295.0580 379<br>297.0772 547<br>313.0706 2249<br>323.0889 437<br>397.0894 580<br>415.1027 965<br>433.1124 2668<br>434.1187 500	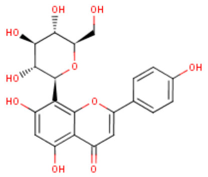 Vitexin $C_{21}H_{20}O_{10}$	[M+H]+	<20	MetaboScape
87	215	8.4	274.2738	282.7	39.0227 664<br>41.0383 660<br>44.0495 542<br>57.0706 1484<br>70.0654 628<br>88.0749 492<br>106.0863 462<br>256.2633 784<br>274.2746 15490<br>275.2781 2832	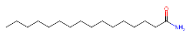 Palmitic amide $C_{16}H_{33}NO$<br><br>Fragments:<br><br>256.2633 784	[M+H]+	<20	MetaboScape
88	216	8.5	318.3000	113.2	41.0394 258<br>43.0546 290<br>44.0500 125<br>45.0338 712<br>57.0701 147<br>70.0656 153<br>256.2647 306<br>318.3008 2394<br>319.3036 410	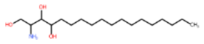 Phytosphingosine $C_{18}H_{39}NO_3$ LMSP01030001<br><br>Fragments:<br><br>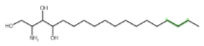 41.0394<br><br>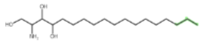 43.0546	[M+H]+	1.42	lipidmaps

No	Peak No	Retention time	Mass	S/N	Fragmentation	Assigned identification	Adduct	Mass Tolerance (ppm)	Database
						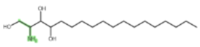<br>44.05<br>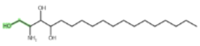<br>45.0338<br>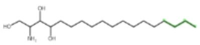<br>57.0701			
89	217	8.5	362.3258	23.5	43.0541 211<br>45.0340 486<br>56.0500 503<br>57.0700 242<br>58.0653 197<br>104.0696 218<br>122.0804 1497<br>300.2884 177<br>362.3270 2285<br>363.3304 474	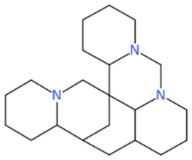<br>(-)-Jamine<br>$C_{21}H_{35}N_3$<br>C10768<br><br>Fragments:<br>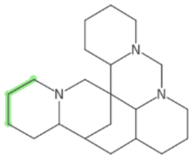<br>43.0541<br>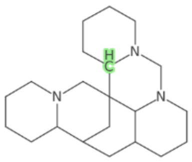<br>45.034	[M+CH3 3OH+H] +	29.15	KEGG

No	Peak No	Retention time	Mass	S/N	Fragmentation	Assigned identification	Adduct	Mass Tolerance (ppm)	Database
						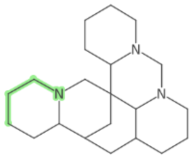<br>56.05<br>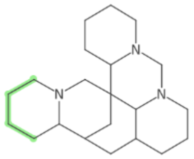<br>57.07<br>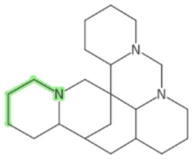<br>58.0653			
90	223	8.78	197.117	36.4	79.0550 1005<br>97.0644 1424<br>125.0597 921<br>143.0697 5948<br>149.0227 921<br>161.0804 35435<br>162.0844 2681<br>183.0629 83686<br>184.0663 6534<br>185.0683 838	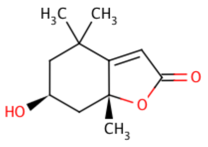<br>Loliolide<br>C11H16O3	[M+H]+	<20	MetaboScape
91	225	8.81	155.0467	49.8	98.9822 321<br>116.9952 110	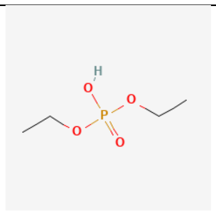<br>O,O-Diethyl phosphate<br>C4H11O4P	[M+H]+	<20	MetaboScape
92	226	8.83	183.078	69.0	55.9345 159<br>56.9427 463<br>62.9636 114<br>80.9734 569<br>98.9835 6885<br>116.9940 1105<br>127.0159 545	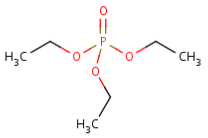<br>Triethyl phosphate<br>C6H15O4P	[M+H]+	<20	MetaboScape

No	Peak No	Retention time	Mass	S/N	Fragmentation	Assigned identification	Adduct	Mass Tolerance (ppm)	Database
93	230	9.0	232.1331	374.3	53.0393 455<br>77.0385 170<br>84.0807 180<br>102.0906 231<br>103.0538 2559<br>104.0565 268<br>131.0488 2931<br>132.0522 225<br>232.1344 819<br>233.1349 147	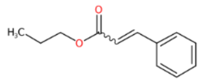<br>Propyl cinnamate<br>$C_{12}H_{14}O_2$<br>C06360<br>Fragments;<br>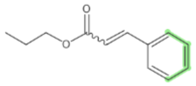<br>53.0393<br>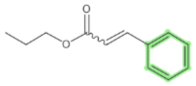<br>77.0385<br>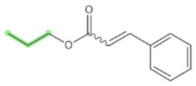<br>84.0807<br>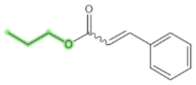<br>102.0906<br>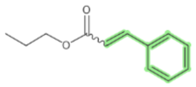<br>103.0538	[M+ACN+H]+	2.05	KEGG

No	Peak No	Retention time	Mass	S/N	Fragmentation	Assigned identification	Adduct	Mass Tolerance (ppm)	Database
						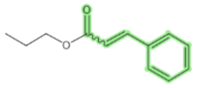<br>131.0488			
94	231	9.0	463.2581	25.4	84.0808 392<br>102.0908 1088<br>103.0534 1646<br>131.0485 20251<br>132.0523 1832<br>214.1216 1123<br>215.1258 168<br>232.1331 26975<br>233.1357 3835<br>234.1397 203	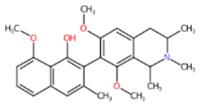<br>Ancistrotectorine<br>$C_{26}H_{31}NO_4$<br>C12340<br><br>Fragments:<br>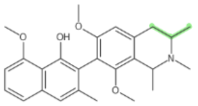<br>84.0808<br>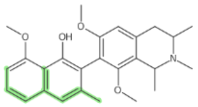<br>103.0534<br>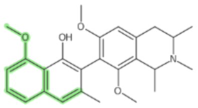<br>131.0485<br>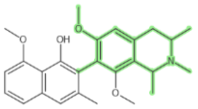<br>214.1216	[M+ACN +H]+	1.45	KEGG

No	Peak No	Retention time	Mass	S/N	Fragmentation	Assigned identification	Adduct	Mass Tolerance (ppm)	Database
						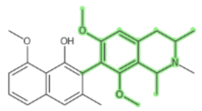<br>232.1331			
95	239	9.1	241.1066	159.8	53.0385 262<br>107.0477 305<br>121.0277 252<br>135.0438 345<br>137.0594 563<br>148.0519 633<br>151.0389 478<br>166.0634 238<br>181.0859 5537<br>182.0887 623	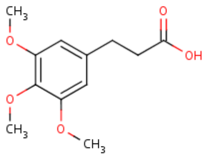<br>3,4,5-Trimethoxydihydrocinnamic acid<br>$C_{12}H_{16}O_5$	[M+H]+	<20	MetaboScape
96	241	9.1	503.1878	37.5	181.0852 126<br>182.0893 194<br>263.0884 8971<br>264.0926 1485<br>265.0982 139<br>281.0977 1976<br>282.1002 253<br>304.1160 231<br>484.2928 138<br>503.1539 170	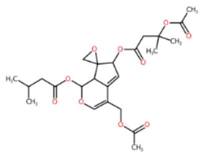<br>Acevaltrate<br>$C_{24}H_{32}O_{10}$<br>C16752<br><br>Fragments:<br>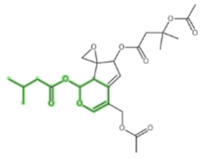<br>181.0852<br>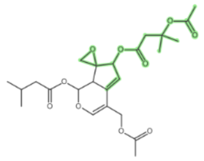<br>263.0884	[M+Na]+	2.25	KEGG
97	242	9.2	276.1227	29.1	43.0182 107<br>53.0390 247<br>103.0534 738<br>131.0490 1809<br>132.0512 177<br>191.0722 281<br>276.1217 108	Cinnamic acid ethyl ester	[M+H]+	<20	Plant Metabolites

No	Peak No	Retention time	Mass	S/N	Fragmentation	Assigned identification	Adduct	Mass Tolerance (ppm)	Database
98	249	9.4	292.1539	47.6	55.0542 201<br>131.0480 169<br>135.0443 156<br>147.0431 340<br>163.0387 292<br>175.0388 124<br>190.0620 187<br>221.0810 1790<br>222.0841 208<br>292.1533 275	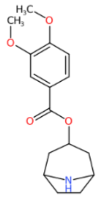<br><br>Convolvine<br>$C_{16}H_{21}NO_4$<br>C10856<br><br>Fragments:<br>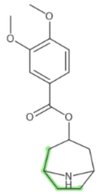<br>55.0542<br>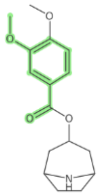<br>135.0443<br>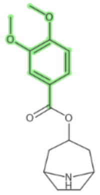<br>147.0431<br>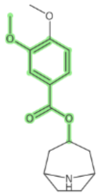<br>163.0387<br>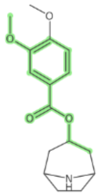<br>175.0388	[M+H]+	1.79	KEGG

No	Peak No	Retention time	Mass	S/N	Fragmentation	Assigned identification	Adduct	Mass Tolerance (ppm)	Database
						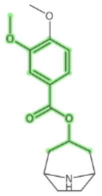<br>190.062<br>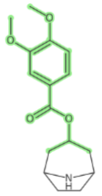<br>221.081			
99	274	10.3	274.1436	168.0	53.0388 241<br>84.0801 110<br>103.0534 2465<br>104.0565 200<br>131.0485 3977<br>132.0522 412<br>144.1015 141<br>214.1226 403<br>274.1447 135	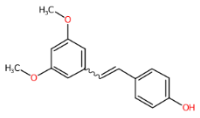<br>Pterostilbene<br>$C_{16}H_{16}O_3$<br>C10287<br><br>Fragments:<br>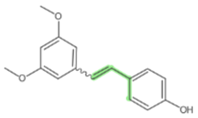<br>53.0388<br>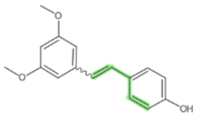<br>84.0801<br>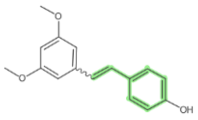<br>103.0534	[M+NH4]+	0.98	KEGG

No	Peak No	Retention time	Mass	S/N	Fragmentation	Assigned identification	Adduct	Mass Tolerance (ppm)	Database
						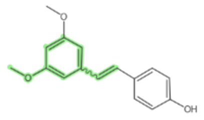<br>131.0485<br>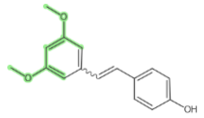<br>144.1015<br>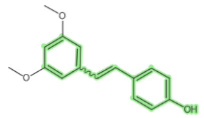<br>214.1226			
100	284	11.1	524.3424	17.4	57.0702 580<br>122.0810 486<br>204.0504 1294<br>222.0605 2430<br>258.0820 605<br>386.3082 377<br>506.3323 6657<br>507.3349 2077<br>508.3329 659<br>524.3421 1103	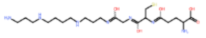<br>Glutathionylspermine<br>$C_{20}H_{41}N_7O_5S$<br>C16562<br><br>Fragments:<br>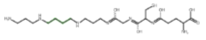<br>57.0702<br>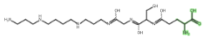<br>122.081	[M+CH3 OH+H]	41.36	KEGG
101	288	10.83	216.1378	52.5	39.0224 286<br>53.0026 236<br>53.0389 1056<br>69.0323 236<br>86.0984 458<br>103.0537 1684	3-Hydroxy-3-phenylpropionic acid	[M+H]+	<20	Plant Metabolites

No	Peak No	Retention time	Mass	S/N	Fragmentation	Assigned identification	Adduct	Mass Tolerance (ppm)	Database
					131.0482 2066				
102	295	11.06	225.1956	24.1	44.0138 433<br>51.0235 450<br>53.0390 1240<br>55.0543 2286<br>58.0652 520<br>61.0398 852<br>100.1126 508<br>143.1167 561<br>165.0538 426<br>225.1951 2406	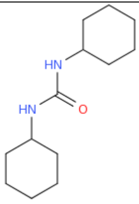<br>1,3-Dicyclohexylurea<br>C13H24N2O	[M+H]+	<20	MetaboScape
103	317	11.7	288.2529	22.4	39.0239 327<br>42.0343 318<br>43.0545 1292<br>45.0343 578<br>57.0702 855<br>70.0647 332<br>88.0759 625<br>106.0857 904<br>152.0625 252<br>227.2001 266	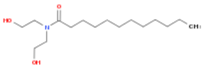<br>N,N-Bis(2-hydroxyethyl)dodecanamide<br>C16H33NO3	[M+H]+	<20	MetaboScape
